# A Probabilistic Model for Indel Evolution: Differentiating Insertions from Deletions

**DOI:** 10.1093/molbev/msab266

**Published:** 2021-09-01

**Authors:** Gil Loewenthal, Dana Rapoport, Oren Avram, Asher Moshe, Elya Wygoda, Alon Itzkovitch, Omer Israeli, Dana Azouri, Reed A Cartwright, Itay Mayrose, Tal Pupko

**Affiliations:** 1 The Shmunis School of Biomedicine and Cancer Research, George S. Wise Faculty of Life Sciences, Tel Aviv University, Tel Aviv, Israel; 2 School of Plant Sciences and Food Security, George S. Wise Faculty of Life Sciences, Tel Aviv University, Tel Aviv, Israel; 3 The Biodesign Institute, Arizona State University, Tempe, AZ, USA; 4 School of Life Sciences, Arizona State University, Tempe, AZ, USA

**Keywords:** molecular evolution, evolutionary models, indels, alignments, approximate Bayesian computation

## Abstract

Insertions and deletions (indels) are common molecular evolutionary events. However, probabilistic models for indel evolution are under-developed due to their computational complexity. Here, we introduce several improvements to indel modeling: 1) While previous models for indel evolution assumed that the rates and length distributions of insertions and deletions are equal, here we propose a richer model that explicitly distinguishes between the two; 2) we introduce numerous summary statistics that allow approximate Bayesian computation-based parameter estimation; 3) we develop a method to correct for biases introduced by alignment programs, when inferring indel parameters from empirical data sets; and 4) using a model-selection scheme, we test whether the richer model better fits biological data compared with the simpler model. Our analyses suggest that both our inference scheme and the model-selection procedure achieve high accuracy on simulated data. We further demonstrate that our proposed richer model better fits a large number of empirical data sets and that, for the majority of these data sets, the deletion rate is higher than the insertion rate.

## Introduction

Insertions and deletions (indels) shape genes and genomes and are fundamental in molecular evolution research (Cartwright 2009). Indels are of great importance for ancestral sequence reconstruction (Ashkenazy et al. 2012; Vialle et al. 2018), and substantially contribute to divergence among species (Britten 2002, 2003; Anzai et al. 2003; Wetterbom et al. 2006). Fitch (1973) was the first to observe that deletions may be more common than insertions, however, this observation was based on very few protein sequences. De Jong and Ryd**é**n (1981) analyzed a much larger set of proteins and suggested that deletions are 4-fold more frequent than insertions and that this phenomenon is an inherent property of the replication mechanism. In support of this hypothesis, Graur et al. (1989) found over three times more deletions than insertions in processed human and rodent pseudogenes, suggesting that mutations rather than selection drive the excess of deletion over insertion events. This deletion bias was confirmed by numerous other studies (Ogata et al. 1996; Petrov et al. 1996; Ophir and Graur 1997; Mira et al. 2001; Zhang and Gerstein 2003; Fan et al. 2007; Van Passel et al. 2007; Kuo and Ochman 2009). Regarding the distribution of indel length, it was repeatedly observed that both in proteins and DNA sequences, single-site indels are the most frequent and the occurrence of indels declines monotonically as a function of their length (Pascarella and Argos 1992; Benner et al. 1993; Golenberg et al. 1993; Gu and Li 1995; Qian and Goldstein 2001). Two distributions were proposed for the indel length: geometric and Zipfian. It was previously shown that the Zipfian distribution better fits biological data sets, both for proteins (Benner et al. 1993) and for noncoding regions (Saitou and Ueda 1994). Gu and Li (1995) found only small differences in the size distribution of deletions and insertions. When insertions and deletions were treated together, the parameter of the Zipfian length distribution varied from 1.70 in primate globin noncoding regions to 1.93 in noncoding mitochondrial DNA. Of note, these early studies were based on small data sets, such that only a few indel events were considered. In another study that analyzed coding and noncoding indels in 18 mammalian genomes, differences were found both among species and between insertions and deletions: The Zipfian parameter ranged from 1.059 to 1.883, when modeling the length distribution of deletions in chimpanzee to insertions in rabbit, respectively (Fan et al. 2007).

In all these studies, the indel parameters were inferred based on gap counts. However, gaps can reflect more than one event, for example, an alignment gap of length 12 can reflect a single insertion event of 12 residues, or many possible combinations involving multiple events, for example, an insertion event of 11 residues followed by another insertion of a single residue, an insertion event of 13 residues followed by a deletion event of a single residue, etc. Counting methods ignore these latter possibilities, similar to parsimony methods that ignore potential multiple substitutions at a single site. Moreover, in previous approaches only gaps which could be reliably inferred among the analyzed sequences were included. Often, overlapping gaps were excluded. Selecting only a subset of gaps which conforms to an ad hoc criterion potentially introduces a bias in the collection of indels analyzed. In addition, the accuracy of indel parameter estimates is expected to be positively correlated with the number of analyzed indel events. Retaining only reliable indels, usually those occurring between closely related sequences, substantially reduces the amount of information available for indel inference. Ignoring a large fraction of indel events is especially problematic when the goal is to compare indel dynamics among specific genes. In this case, the number of gene-specific indel events is limited, and discarding all unreliable indels from the analysis is expected to lead to poor performance of indel inference approaches. These concerns call for probabilistic-based methods for indel parameter inference.

Probabilistic-based models for indels are far less developed compared with substitution models. This might be the case because indel models violate the assumption of site independence, thus complicating the computation of the likelihood function (Cartwright 2005; Fletcher and Yang 2009). More elaborate methodologies to estimate indel parameters include Cartwright’s lambda.pl Perl script released with the DAWG simulation package (Cartwright 2005). It assumes a Poisson distribution for indel rates and estimates the distribution using the maximum-likelihood paradigm. The method uses linear regression to find the best-fitted Zipfian distribution for the indel length and takes the average length of the input sequences as the root length. Two additional methods are based on hidden Markov model (HMM) between pairs of divergent sequences (Lunter 2007; Cartwright 2009). In Lunter (2007), biases introduced by alignment programs were explicitly accounted for, for example, the tendency of alignment algorithms to merge two independent gaps (“gap attraction”). In addition, gap lengths were assumed to follow a mixture of geometric distributions. Cartwright (2009) used expectation maximization algorithm based on a pairwise HMM for the inference of model parameters. This method assumes independence between indel events and ignores overlapping indels. These methods were restricted to pairwise sequences, and thus could not distinguish between insertion and deletion rates. Mikl**ó**s et al. (2004) developed a full probabilistic evolutionary Markov model that includes substitutions, insertions, and deletions. This “long-indel model” is both context-independent and time-reversible, and indel lengths are assumed to follow a geometric distribution. Despite the introduction of efficient means to accelerate computations with the long-indel model (Levy Karin et al. 2019), it is still computationally intensive, and inference using this model is currently limited to pairwise sequences.

Keeping the benefit of probabilistic-based approaches without falling to the computational hurdles of likelihood-based methods for inferring indel parameters, previously motivated us to develop SPARTA (Levy Karin et al. 2015), an algorithm to learn indel parameters from input MSAs based on probabilistic simulation of alignments. SPARTA is an ad hoc methodology that is not rooted in statistical inference theory. We later developed Sparta ABC (Levy Karin et al. 2017), which is based on the approximate Bayesian computing (ABC) methodology, a statistically rigorous methodology for the inference of model parameters. The ABC framework, first introduced in molecular evolutionary studies for population genetics (Beaumont et al. 2002), has been utilized successfully to estimate parameters in complex models in which the likelihood function is challenging to compute. ABC was successfully employed, for example, for estimation of the effective population size from a sample of microsatellite genotypes (Tallmon et al. 2008), for estimation of divergence times and admixture by analyzing whole genomes of chimpanzee and bonobo populations (Kuhlwilm et al. 2019), and for inference of relevant parameters relating to selective sweeps; that is, selection coefficient, time of selection onset, recombination rate, and mutation rate at neutral loci (Przeworski 2003). ABC methodologies thus retain the benefits of analyzing data with explicit probabilistic models, yet overcome computational limitations of inference schemes that rely on explicit inference of the likelihood function.

The underlying indel probabilistic model in SpartaABC assumes that the insertion rate (number of insertion events per substitution event) equals the deletion rate. It further assumes that the length of an insertion (number of newly introduced nucleotides or amino acids) has the exact same distribution as the length of a deletion. As stated above, these assumptions are known to be an oversimplification of indel dynamics. In this study, we develop a more realistic alternative by assigning different parameters for insertions and deletions. We also apply a model-selection scheme to determine whether the richer model better describes indel evolutionary dynamics compared with the simpler one. Our results demonstrate that the richer model fits a large number of empirical biological data sets, lending further statistical support for the hypothesis that deletions are more common than insertions.

## New Approaches

### Indel Models

We describe two indel models, a simple indel model (SIM) and a rich indel model (RIM), which alleviates some of the assumptions made in SIM. The parameters of both models are summarized in table 1. In SIM, insertions and deletions are assumed to have the same rates and length distributions. Thus, SIM has three parameters: 1) indel-to-substitution-rate ratio (*R_ID*). Note that this parameter quantifies the sum of the insertion and the deletion rates, assumed to be equal in this model. 2) The insertion length distribution parameter (*A_ID*), which dictates the lengths distribution of newly inserted or deleted segments. Qian and Goldstein (2001) showed that the frequencies of indels that are several dozens of amino acid long are lower than their expected frequencies, when the expectation is computed based on the length distribution of shorter indels. Thus, in our models, it is assumed that indels length is distributed as truncated Zipfian (power law) with maximum indel size of 50 amino acids and a rate parameter *A_ID* (*A_ID* stands for the “*a*” parameter of the Zipfian distribution for insertion/deletion length). 3) The sequence length at the root of the tree (RL). In RIM, different indel parameters are assigned to insertions and deletions, resulting in five free parameters. In addition to the root length, two parameters dictate the indel rates, one for insertions (*R_I*) and one for deletions (*R_D*). Similarly, two “*a*” parameters are assumed, one dictating size distribution for insertions (*A_I*) and one for deletions (*A_D*).

**Table 1.** The Simple Indel Model (SIM) and Rich Indel Model (RIM) Parameters and Their Description.

Model	Parameter Name	Description
SIM, RIM	*RL*	The sequence length at the root of the tree
SIM	*R_ID*	Indel-to-substitution-rate ratio
	*A_ID*	Indel length distribution parameter
RIM	*R_I*	Insertion-to-substitution-rate ratio
	*R_D*	Deletion-to-substitution-rate ratio
	*A_I*	Insertion length distribution parameter
	*A_D*	Deletion length distribution parameter

### Prior Distributions of Model Parameters

Model parameters are inferred using ABC. In this Bayesian inference scheme, prior distributions over model parameters have to be chosen. We assume the following prior distributions: 1) The indel to substitution rates are assumed to be uniformly distributed in the range [0, 0.1] for *R_ID* (SIM) and [0, 0.05] for *R_I and R_D* (RIM). 2) The parameters that dictate the indel length distribution (*A_ID* for SIM, and *A_I and A_D* for RIM) are assumed to be uniformly distributed in the range [1.001, 2]. 3) The RL parameter range is determined according to the input sequences, as follows: Let $l_{s}\;\mathrm{and}l_{l}$ be the length of the shortest and longest sequences, respectively, of the unaligned sequences, then the range of RL is assumed to be uniformly distributed in the range [0.8$l_{s}$,1.1$l_{l}$]. We note that increasing the range of the prior distributions had little effect on the results (not shown).

### Inference Outline (without Accounting for Alignment Uncertainty)

The ABC inference scheme relies on several components/steps: 1) generating simulations; 2) computing summary statistics; 3) assigning summary statistics weights; 4) accepting a subset of the simulations; and 5) inferring the posterior distributions and point estimates. These components are described in detail below. Here, we first present a general outline of the algorithm. The input required to infer the model parameters for a data set in question is a multiple sequence alignment (MSA) and its associated (rooted) phylogenetic tree, including the topology and its associated branch lengths. Next, a large set of MSAs is generated, by repeatedly simulating the evolutionary process along the input phylogenetic tree, with model parameters sampled from the prior. Next, summary statistics are computed for both the input MSA and each of the simulated MSAs. Summary statistics weights are next computed from a subset of these simulations and are then used to compute distances between the summary statistics of the input MSA and each of the simulated MSAs. A small subset of simulations, for which the distance is very small, is kept. Intuitively, the kept simulations resemble the input data in terms of indel dynamics and can be used to get a point estimate of the model parameters of the data set in question. The distribution of model parameters used to generate this subset is a good approximation for their posterior distribution (Sisson 2018). Thus, the last step of the algorithm is to infer posterior distribution and point estimate for all model parameters by averaging the parameters of the accepted simulations (Tavar**é** et al. 1997).

### Inference: Accounting for Alignment Uncertainty

**Fig. 1.**
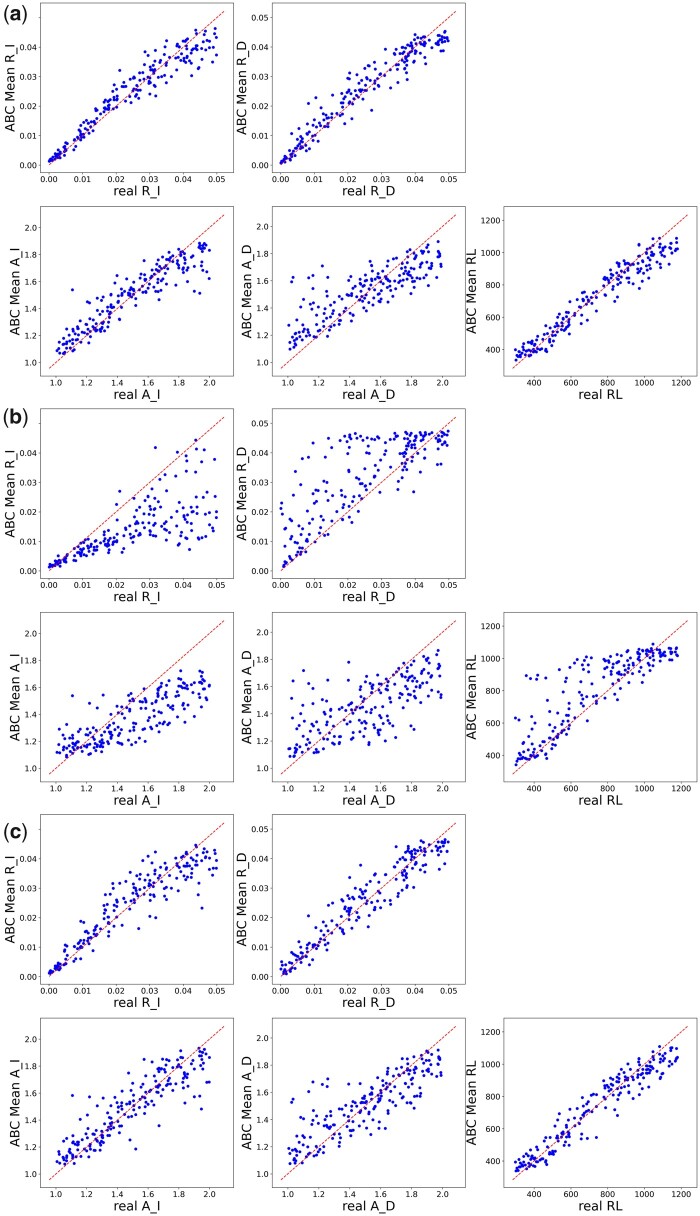
Model accuracy in simulations: inferred parameters (R_I, R_D, A_I, A_D, and RL) are correlated to the parameters used for simulation. Each point represents a single simulation inference for the corresponding parameter against the real value. Each graph is based on 200 independent simulations. The dashed line is the identity y=x line. (*a*) The performance when the input alignment is the true alignment, that is, the performance in ideal settings in which the input alignment is error free. The obtained $R^{2}$ values are 0.93, 0.93, 0.86, 0.69, and 0.94 for R_I, R_D, A_I, A_D, and RL, respectively. (*b*) The performance without correcting for biases introduced to the empirical alignment (i.e., although the summary statistics of the analyzed alignment were derived from MAFFT alignments, the summary statistics of the simulated alignments within the ABC inference were inferred based on alignments not inferred using MAFFT). In each case, the true alignment was unaligned and realigned using MAFFT. The obtained $R^{2}$ values are 0.55, 0.64, 0.67, 0.49, and 0.75 for R_I, R_D, A_I, A_D, and RL, respectively. (*c*) The summary statistics of the simulated alignments within the ABC inference scheme were corrected for biases introduced by MAFFT. The obtained $R^{2}$ values are 0.87, 0.92, 0.81, 0.71, and 0.93 for R_I, R_D, A_I, A_D, and RL, respectively.

In the above inference scheme, the analyzed empirical alignment is computed using alignment inference tools such as MAFFT (Katoh and Standley 2013). However, the simulated alignments that are generated as part of the ABC procedures are “true” alignments and are not inferred. It is possible that this discrepancy introduces a bias in the inference. We validated that such a bias indeed exists: We simulated an empirical alignment for which we know the “true” indel parameters and inferred the parameters using the above described ABC inference method. We then unaligned the “true” alignment, re-aligned it with MAFFT, and repeated the above ABC inference procedure. The results clearly show that the performance of the inference scheme is substantially reduced when MAFFT-based alignments, rather than “true” alignments, are provided as input (fig. 1*a and b*).

One possible solution for correcting this bias would be to realign each simulated data set (i.e., remove all gaps and apply MAFFT on the unaligned sequences) within the ABC inference scheme. This, however, would make the inference procedure enormously CPU intensive. Hence, we tested an alternative approach: We use a machine-learning-regression algorithm to learn how MAFFT distorts each of the summary statistics. We then corrected each summary statistics of each simulated alignment within the ABC inference scheme. More specifically, given an empirical MSA, its corresponding phylogenetic tree, and a model (SIM or RIM), we first generated 200 simulated MSAs, in which model parameters were sampled from the prior distribution (see explanations about how sequences are simulated below). In the learning phase, which is done separately for each empirical data set analyzed, we computed the summary statistics for each simulated MSA. Next, we realigned (using MAFFT) the 200 alignments and recomputed the summary statistics. Our goal is now to compute a regression model for each summary statistic. This is done by computing a multivariate regression using as predictors the set of 27 summary statistics before the alignment procedure, as well as the model parameters (three for SIM and five for RIM). The response variable of each of these regressions is the value of the summary statistics after the alignment procedure. Thus, 27 regressions are computed, one for each summary statistics. For RIM, each regression is thus from ℛ^32^ to ℛ, where ℛ denotes real numbers. An example of summary statistics derived from a simulated data before and after this correction are provided in supplementary table S1, Supplementary Material online. To avoid potential overfitting, the regression curves are computed using Lasso (Tibshirani 1996) with 3-fold cross-validation to determine the regularization parameter. Depending on the data analyzed, the inclusion of some summary statistics may introduce more noise than signal. To this end, for each summary statistics, we computed the Pearson correlation coefficient between its values following MAFFT alignments and the inferred value based on the regression model. We excluded summary statistics for which the correlation coefficient was less than 0.9.

### Simulator

Existing tools for simulating sequences such as DAWG 2.0 (Cartwright 2005) and INDELible (Fletcher and Yang 2009) account for both substitution and indel events. For the purpose of inferring the relevant summary statistics, the information regarding substitutions can be ignored. Thus, simulations can be performed without substitutions, thereby reducing simulation running times, which are a major component of the ABC inference scheme. To this end, we developed an indel simulator for SIM and RIM that ignores substitution events. We implemented such a simulator in C++ following the Gillespie algorithm (Gillespie 1977). In essence, we first draw model parameters from the prior, which also provide the length of the root sequence. Then, along each branch, the *R_I and R_D* parameters dictate the waiting time for either an insertion or a deletion event as described in INDELible (Fletcher and Yang 2009). Once an event has occurred, the indel length is drawn from a Zipfian distribution with rate parameter *A_I and A_D* for insertions and deletions, respectively. The location of indels is next drawn uniformly based on the sequence length at the time the event has occurred. We introduce a correction for indels at the boundaries of the sequence. Specifically, assume we draw a deletion of length five. Now we draw the location uniformly, from position −5 to *L*, where *L* is the length of the current sequence. If for example, the position is −1, we delete the first four characters of the sequence. By doing so, it is guaranteed that the boundaries do not distort the uniform rate of indel events within the sequence (otherwise, deletion in the 5′ site for nucleotide sequences or the N terminus for proteins would have been underrepresented). If the next event occurs at a time which is longer than the branch length, we ignore this event, and set the sequence in the next node to be identical to that of the current sequence. Once the sequences of all leaves are generated, based on the record of all indel events along the tree, the simulated MSA is constructed. A detailed explanation of how simulations are generated is provided in supplementary figure S1, Supplementary Material online.

For studying the distortion of summary statistics introduced by alignment algorithms such as MAFFT, sequences including substitutions must be generated. Only for these alignments, we use the following procedure for simulating the alignments: 1) an alignment without substitutions is generated as described above; 2) an alignment without indels, and with the length of the alignment in (1), based on the same tree, is generated using INDELible. For this, we use either a nucleotide or amino acid substation model *(*GTR+I + G [Abadi et al. 2019] or WAG [Whelan and Goldman 2001], respectively); 3) we superimpose the two alignments (supplementary fig. S1, Supplementary Material online).

### Summary Statistics

The 27 summary statistics calculated in the inference scheme are described in table 2. This list extends the 11 summary statistics previously used by Levy Karin et al. (2017), which included for example the 10th and the 11th summary statistics, that is, the minimum and maximum length of sequences in the alignment, respectively. Such summary statistics are influenced by all model parameters, they strongly vary depending on the indel rates, the distribution of indel lengths, and the root length. New summary statistics were introduced to help differentiate insertion from deletion events. For example, the 13th summary statistic, that is, number of MSA columns that contain a single gap, provides information on deletion rates, as a column with a single gap typically reflects a single deletion event. Another example is the 18th summary statistic, which counts the number of MSA columns in which a single-residue gap is found in all but one sequence. Such a column likely reflects an insertion of a single residue in a branch leading to a leaf of the tree. Notably, such a column may result from a deletion event as well. The ABC approach does not assume that this is certainly an insertion event, but rather, all summary statistics are considered together and their values provide information regarding the posterior probability of the model parameters. We provide an example of a simulated alignment and its associated summary statistics in supplementary figure S1 and table S1, Supplementary Material online.

**Table 2.** The 27 Summary Statistics Used in the ABC Scheme.

No.	Summary Statistics
1	Total number of gap blocks in the alignment
2	Total number of unique gap blocks in the alignment
3	Average gap block length
4	Average unique gap block length
5	Number of gap blocks of length one
6	Number of gap blocks of length two
7	Number of gap blocks of length three
8	Number of gap blocks of length four or more
9	Alignment length
10	Minimum length of sequence in the alignment
11	Maximum length of sequence in the alignment
12	Number of MSA columns with zero gap
13	Number of MSA columns with one gap
14	Number of MSA columns with two gaps
15	Number of MSA columns with n-1 gaps
16	Number of gaps of length one that appear only in one sequence
17	Number of gaps of length one that are shared between exactly two sequences
18	Number of gaps of length one that are shared between exactly n-1 sequences
19	Number of gaps of length two that appear only in one sequence
20	Number of gaps of length two that are shared between exactly two sequences
21	Number of gaps of length two that are shared between exactly n-1 sequences
22	Number of gaps of length three that appear only in one sequence
23	Number of gaps of length three that are shared between exactly two sequences
24	Number of gaps of length three that are shared between exactly n-1 sequences
25	Number of gaps of length at least four that appear only in one sequence
26	Number of gaps of length at least four that are shared between exactly two sequences
27	Number of gaps of length at least four that are shared between exactly n-1 sequences

### Computing Weights for the Summary Statistics

Let $D_{i}$ and $D_{s}$ denote an input MSA and a simulated MSA, respectively. Let $S(D_{i})$ and $S(D_{s})$ be summary statistics vectors associated with $D_{i}$ and $D_{s}$, respectively. In order to decide whether or not to keep a simulation, a weighted Euclidean distance is computed between $S(D_{i})$ and $S(D_{s})$ as follows:

$$d\left(S\left(D_{i}\right),S\left(D_{s}\right)\right)=\sqrt{\sum_{j=1}^{27}\omega_{j}{\left(S{\left(D_{i}\right)}_{j}-S{\left(D_{s}\right)}_{j}\right)}^{2}}$$

where the subscript j is the summary statistics index and $\omega_{j}$ denotes the weight of the *j*th summary statistic. The various summary statistics differ in their magnitude, so different weights are required to ensure that all the summary statistics contribute approximately equally to the distance. Hence, the weight of each summary statistics is set as $\omega_{j}=\frac{1}{\hat{\sigma_{j}^{2}}}$, where $\hat{\sigma_{j}}$ is the estimated standard deviation of the *j*th summary statistics across B simulations with indel parameter values drawn at random from the prior. We set B=10,000, because for this value, the vector of weights practically converged in all cases (not shown).

### Acceptance/Rejection Criterion

The weighted Euclidian distance is calculated for *N_s_* simulations. By default, *N_s_* = 100,000 (using 1,000,000 simulations did not significantly improve the performance; not shown**)**. The set of accepted simulations are chosen such that the rate of accepted simulations is *p* of the total simulations (Beaumont et al. 2002**)**. In this study, the *p* parameter was set to 0.1% (100/100,000**)** of the simulations (0.1% yielded the best performance in a small-scale simulation study, not shown).

## Results

### Inference Accuracy on Simulated Data

We tested the accuracy of SpartaABC in inferring model parameters by simulating data sets with model parameters sampled from the prior based on a specific tree topology and an MSA sampled from the EggNOG database (Huerta-Cepas et al. 2019). The MSA contains 129 sequences, with a mean sequence length of 817 amino acids (data set: ENOG504B73R). To quantify inference accuracy, we computed the $R^{2}$ values between the true parameters and the inferred ones, over 200 random different parameter combinations sampled from the prior distribution. The obtained $R^{2}$ values were 0.87, 0.92, 0.81, 0.71, and 0.93, for *R_I, R_D, A_I, A_D*, and *RL*, respectively (fig. 1*c*). We extended this simulation analysis by repeating the simulation scheme for 12 additional data sets that differ from the one presented in figure 1*c* with respect to tree topologies, total branch lengths, number of species, and sequence length (supplementary table S2, Supplementary Material online). These simulations demonstrate that the estimates of the parameters controlling the indel rates and root length (*R_I, R_D*, and *RL*) are more accurate than those dictating the length distribution of indels (*A_I* and *A_D*). The inference accuracy strongly increases as a function of the total branch lengths (supplementary fig. S2, Supplementary Material online). Our results further suggest that SpartaABC provides relatively unbiased estimates for *R_I, R_D* and *RL*, whereas it tends to underestimate *A_I* and *A*_*D*, for which the slope of the regression fit is smaller than 1.0 (supplementary table S2, Supplementary Material online). We conclude that SpartaABC provides accurate estimates of model parameters, most notably for the indel rates and the root length, as long as sufficient indels have accumulated to allow reliable inference.

### Feature Importance

We use the terms “features” and “summary statistics” interchangeably. The impact of each summary statistics on the inference accuracy of SpartaABC was examined using simulations. SpartaABC computes 27 summary statistics for the input MSA and for each simulated MSA (table 2). The importance of each summary statistics for the inference accuracy of each of the five inferred parameters can be obtained by comparing the performance with all features versus the performance when a specific feature is excluded from the analyses. Notably, a certain feature may be important for the inference of one parameter, but unimportant for another. Moreover, some variability of feature importance is expected, to a certain degree, among data sets.

We computed a feature-importance score for each summary statistics for each of the five RIM parameters (supplementary fig. S3, Supplementary Material online). The most important features for root length estimate were the shortest and the longest sequences, respectively. Regarding the rate parameters, although there are no substantial differences in feature importance regarding the *R_I* parameter, for *R_D*, the most important feature was the number of alignment columns with only a single gap, which is expected as this feature is highly associated with deletion events. For the *A_I* parameter, which dictates the size distribution of newly inserted sequences, the most important summary statistics was the number of gap blocks of length one, and the second most important summary statistics was the number of alignment columns that are all gaps but a single sequence. This last feature is highly associated with novel insertion events. For the *A_D* parameter, which dictates the size distribution of new deletion events, the most important summary statistics was the number of gaps of length one that appear only in one sequence column and the average size of unique gaps. The feature importance analysis demonstrates the benefit of using multiple features for the accurate inference of parameters used in indel models.

### Model Selection

To compare the fit of different models, such as the SIM and RIM described above, with an empirical data set, model selection is needed. We follow the standard ABC model selection approach, in which we sample uniformly from the models (which is equivalent to assuming uniform prior over the models), pool all the simulations and select those that are closest to the empirical data, as defined by the distance threshold. The estimated posterior probability of each model is approximated by the relative frequency of retained simulations generated from each model (Pritchard et al. 1999). It was previously shown that ABC model selection can be problematic under some scenarios (Robert et al. 2011), and hence the performance of model selection procedures must be extensively tested using simulations.

**Fig. 2.**
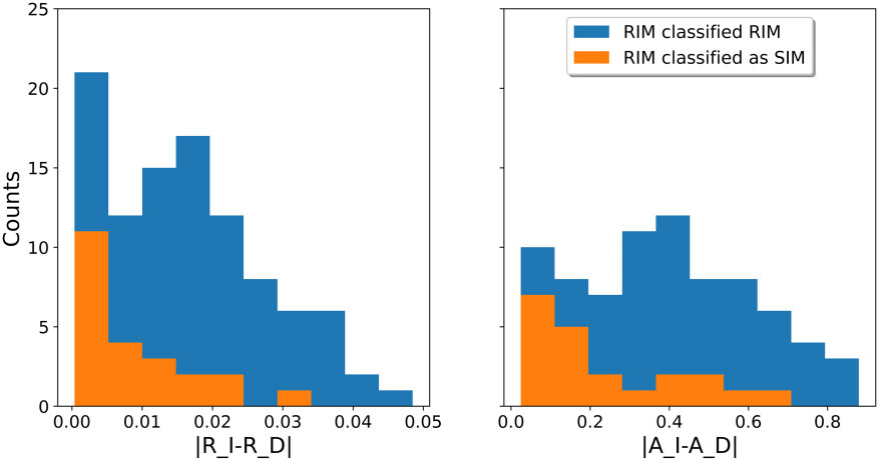
Misclassification rates depend on the similarity between insertion and deletion parameters. The errors depend on the absolute difference between R_I and R_D and the differences between A_I and A_D. All simulations were under the RIM model. In dark, simulations that were correctly classified as RIM and in light, cases which were misclassified as SIM. Results are based on 200 simulated alignments: 100 simulated with the RIM model and 100 simulated with the SIM model. Sequences were simulated based on the topology of the ENOG504B73R data set.

We evaluated the accuracy of the model selection approach using simulations. To this end, we simulated 2,600 data sets (100 MSAs under various SIM parameters, as well as 100 MSAs under various RIM parameters along 13 different trees derived from 13 empirical data sets, see Materials and Methods). The classification accuracy for the ENOG504B73R data set is shown in table 3. For this data set, when the true model was SIM, the model-selection tests had high classification accuracy (98%). When the true model was RIM, the model-selection tests had 77% classification accuracy. These simulation results indicate that the model-selection test slightly favors SIM over RIM making the inference of RIM conservative. Of note, when simulating under RIM, the extent of the differences between the insertion and deletion parameters highly influenced the selected model. Indeed, the model-selection error was strongly dependent on both the difference between *R_I and R_D* and the difference between *A_I* and *A_D* (fig. 2). The mean absolute difference between the *R_I and R_D* parameters for RIM simulations that were correctly classified as RIM was 0.018, whereas when the RIM simulations were misclassified as SIM, it was 0.009 (*t*-test, *P* < 1e-4). The mean absolute difference between the *A_I and A_D* parameters for correctly classified RIM simulations was 0.39, whereas for RIM simulations misclassified as SIM, it was 0.22 (*t*-test, *P* < 2e-2).

**Table 3.** Model Selection Accuracy.

Simulated Model	Accuracy
SIM	0.98
RIM	0.77

Note.—Accuracy is computed based on 100 simulations for each indel model. For example, out of 100 MSAs simulated under RIM, the model selection approach correctly identified 77 as RIM.

We repeated this analysis for 12 additional data sets with various sequence lengths and total branch lengths (supplementary table S3, Supplementary Material online). Similar to the parameter inference accuracy, the model-selection test accuracy also depended on the total branch lengths (supplementary fig. S4, Supplementary Material online).

### Running Times

The average running time for an empirical data set was around 328 min on a single processor, including all simulations, alignments, extraction of features, and model selection between SIM and RIM. The running times for typical data sets are correlated to a linear combination of total branch lengths and number of species of the examined phylogeny ($R^{2}=0.73$, *P* < 2e-4**)**: The running time in minutes is about 36 times the total branch length plus 3 times the number of sequences in the phylogeny. The branch lengths are measured in number of substitutions per site (supplementary fig. S5, Supplementary Material online).

### Empirical Data Analysis

We applied the model selection and inference algorithm on 4,823 biological data sets. These data sets included phylogenetic trees and protein MSAs of various phylogenetic groups including bacteria, plants, insects, fungi, and mammals. Table 4 details the model-selection classifications for various groups. Our method classified the model as RIM for 35% of the examined data sets. The proportions of data sets from which RIM was selected over SIM were similar between prokaryotic and eukaryotic organisms: 34% and 36%, respectively (table 4). The percentage of data sets for which RIM was selected was lowest for Primates (8.33%) and Rodentia (15.19%) and highest for Saccharomycetaceae, *Tenericutes*, and Drosophilidae (51.61%, 49.47%, 48.2%, respectively). We note that it is likely that some of these differences do not reflect genuine differences among taxonomic groups, but rather, differences in the data attributes (supplementary table S4, Supplementary Material online). For example, the average MSA length in each group varies from 661.7 amino acids in *Escherichia* to 2395.4 amino acids in Primates. In addition, differences in the level of sequence divergence exist among the groups, for example, the average sum of branch lengths in Drosophilidae is 3.1 amino acid replacements per site whereas in Primates, it is 1.9 amino acid replacements per site.

**Table 4.** Model Selection for Various Taxonomical Groups.

	Group	No. of SIM	No. of RIM	Percentage of RIM
Prokaryotes	*Bacillus*	147	120	44.94
	*Escherichia*	106	41	27.89
	*P. aeruginosa*	185	88	32.23
	*Rhizobiaceae*	189	81	30.00
	*Staphylococcaceae*	309	85	21.57
	Tenericutes	144	141	49.47
	Vibrionales	263	144	35.38
Eukaryotes	Brassicales	219	43	16.41
	Chlorophyta	291	122	29.54
	Ciliophora	176	156	46.99
	Drosophilidae	245	228	48.20
	Primates	66	6	8.33
	Rhabditida	288	114	28.36
	Rodentia	201	36	15.19
	Saccharomycetaceae	285	304	51.61

**Table 5.** Model Parameters across Various Taxonomical Groups for Protein Data Sets Classified as (*a*) RIM and (*b*) SIM.

Group	RL	R_I	R_D	Mean Insertion Length	Mean Deletion Length
(*a*) RIM					
*Bacillus*	630.7	0.0142	0.0216	6.74	7.65
*Escherichia*	425.0	0.0116	0.0201	5.64	6.40
*P. aeruginosa*	771.9	0.0101	0.0185	6.47	6.72
*Rhizobiaceae*	751.4	0.0101	0.0152	6.49	6.59
*Staphylococcaceae*	591.6	0.0113	0.0180	6.34	7.26
Tenericutes	788.3	0.0103	0.0160	6.19	6.17
Vibrionales	661.2	0.0123	0.0180	6.49	7.36
Brassicales	1395.6	0.0177	0.0364	7.14	8.70
Chlorophyta	800.7	0.0206	0.0266	8.29	8.30
Ciliophora	905.6	0.0198	0.0248	7.98	7.95
Drosophilidae	1826.7	0.0141	0.0390	6.34	7.87
Primates	1376.2	0.0193	0.0344	7.01	7.49
Rhabditida	799.2	0.0235	0.0345	7.59	7.93
Rodentia	1113.1	0.0154	0.0374	6.59	8.70
Saccharomycetaceae	869.5	0.0084	0.0103	6.64	5.51

**Table:** 

Group	RL	R_ID	Mean Indel Length
(*b*) SIM			
*Bacillus*	605.5	0.0468	9.17
*Escherichia*	492.1	0.0251	7.26
*P. aeruginosa*	776.0	0.0187	7.20
*Rhizobiaceae*	766.0	0.0221	7.88
*Staphylococcaceae*	570.2	0.0157	7.25
Tenericutes	692.9	0.0328	7.89
Vibrionales	658.2	0.0287	8.41
Brassicales	795.0	0.0493	9.35
Chlorophyta	677.2	0.0617	10.10
Ciliophora	650.0	0.0629	9.89
Drosophilidae	1428.0	0.0581	8.67
Primates	1091.4	0.0384	8.80
Rhabditida	578.6	0.0557	9.39
Rodentia	834.3	0.0512	9.39
Saccharomycetaceae	1090.2	0.0127	6.54

The mean values of the various model parameters, per taxonomic group, for the RIM and SIM selected data sets are shown in tables 5*a* and 5*b*, respectively. For brevity, the mean insertion and deletion lengths are given instead of the power law parameters. Noticeably, the average *R_D* was higher than the average *R_I* for all examined taxonomic groups (table 5*a*). However, in specific data sets, *R_I* was higher than *R_D*. Specifically, in Saccharomycetaceae, in 56% of the data sets, *R_I* was higher than *R_D*. In Drosophilidae and Rodentia, the deletion rate was approximately twice as high as the insertion rate. However, although in Drosophilidae, about half of the data sets were classified as RIM, the number of data sets for which RIM is supported within Rodentia is only 15.19% (table 4).

**Fig. 3.**
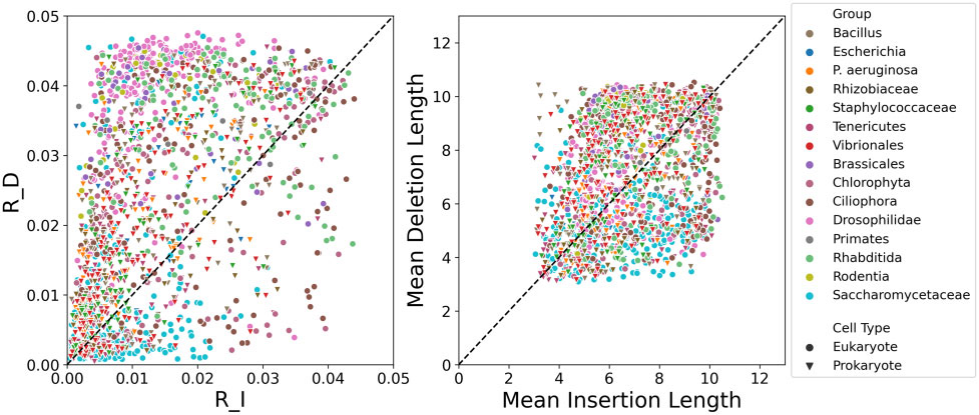
Deletion rates are mostly higher than insertion rates, whereas no significant trend is found for the length distribution. Left panel: a scatter plot of insertion rate (R_I) versus deletion rate (R_D). Right panel: a scatter plot of mean insertion length versus mean deletion length. A total of 1,709 protein data sets across 15 taxonomic groups for which the RIM model was selected are included in the analysis. The dashed line is the identity line, y=x, in both panels.

Figure 3 shows scatter plots of *R_D* versus *R_I* and mean deletion length versus mean insertion length for the data sets classified as RIM. In most of these data sets (1,259 out of 1,709), the deletion rate was higher than the insertion rate. The mean deletion length tended to be higher than the insertion length, however, this trend is quite insubstantial.

**Fig. 4.**
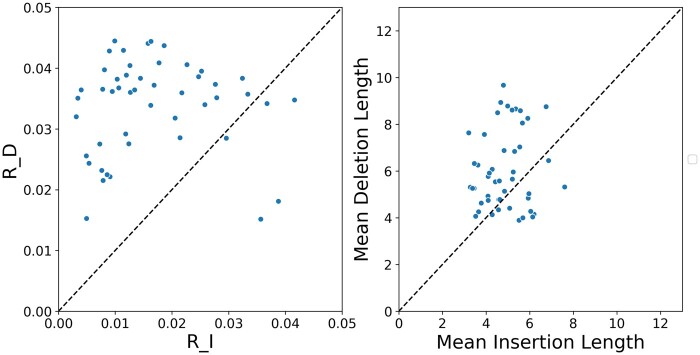
Indel dynamics for empirical DNA sequences. Left panel: a scatter plot of insertion rate (R_I) versus deletion rate (R_D). Right panel: a scatter plot of mean insertion length versus mean deletion length. For the 81 yeast intron data sets analyzed, the number of species ranged from 4 to 7. Shown are results for the 59% data sets for which the RIM model was selected. The dashed line is the identity line, y=x, in both panels.

We next aimed to analyze noncoding empirical data sets. To this end, we applied SpartaABC on 81 intron MSAs from the Yeast Intron DataBase (YIDB) (Lopez and S**é**raphin 2000). The phylogenetic tree topology for the yeast species was taken from the UCSC web browser (Cliften et al. 2003). The branch lengths for each MSA were optimized using RAxML-NG (Kozlov et al. 2019) with the GTR+I + G model (Tavar**é**^1986^; Shoemaker and Fitch 1989; Yang 1994). The GTR+I + G model with the optimized parameters for each data set was also used for the simulations conducted to learn the distortion introduced by MAFFT. Among these data sets, 48 were classified as RIM, whereas 33 were classified as SIM. Among the RIM data sets, the deletion rate was higher than the insertion rate in 89.5% of the data sets (fig. 4, left panel). The average values for the R_D and R_I parameters are 0.034 and 0.017, respectively, that is, a deletion-to-insertion-ratio of 2 (this ratio reduces to 1.5 when the SIM classified data are included). For these data sets, the mean deletion length was 6.1 bp (5.7 bp when the SIM-classified data are included), slightly higher than the mean insertion length, which is 4.8 bp (5.0 bp when the SIM classified data are included) (fig. 4, right panel). These results suggest that differences between insertion and deletion dynamics are also common among non-coding sequences.

## Discussion

In this work, we developed an indel model that accounts for differences between insertion and deletion evolutionary dynamics. Furthermore, we developed an ABC inference scheme to estimate model parameters and to perform a model-selection test that can determine which model (RIM or SIM) better fits a given empirical data set. Additionally, we developed a machine-learning-based step that corrects potential biases introduced by alignment programs. Using simulations, we showed that both the model selection and the inference steps are accurate. Applying the developed inference scheme on a variety of empirical data sets allowed us to gain further insights on indel dynamics. For 35% of the examined protein data sets, the dynamics of insertions and deletions were different. Among these data sets, the deletion rate was higher than the insertion rate in 74%, and to a much lesser extent, the deletion length was larger than the insertion length (55% of these data sets).

Of the analyzed groups, Drosophilidae stands out as the one with the highest deletion rate. It also has a very high fraction of RIM-classified data sets. This finding complies with a previous study that reported exceptionally high deletion rates in *Drosophila* (Petrov et al. 1996). When analyzing yeast intron alignments, insertions and deletions dynamics were different in 59% of the data sets analyzed. Similar to protein coding genes, and even more so, the deletion rate was higher than the insertion rate (90% of the data sets). These results corroborate previous findings regarding the excess of deletions over insertions in both coding and noncoding regions.

Despite this clear overall deletion bias, cases in which the insertion rate is higher than the deletion rate do exist. For example, for Saccharomycetaceae protein-coding genes, although the average deletion rate is higher, the insertion rate was higher than the deletion rate in 56% of the RIM-supported data sets. This is in contrast to only 10% of such cases in yeast introns. Assuming that indel mutations are similar in coding and noncoding regions, the difference between coding and noncoding preference for deletions in yeast implies that selection on indel dynamics highly depends on the genomic context. Recently, Liu and Zhang (2019) provided empirical data suggesting that in *Saccharomyces cerevisiae*, insertion mutations are more common than deletion mutations. Taken together, this implies a very high selection against insertions in yeast introns.

When inferring indel parameters, one expects that as more diverged sequences are analyzed, the alignment will be less reliable, include more misplaced indels, and thus, the inference of indel model parameters may become less accurate. However, the total number of indel events is positively correlated with the level of sequence divergence, suggesting that the accuracy of indel model parameter inference may increase with sequence divergence. Our results (supplementary fig. S2, Supplementary Material online) indicate that within the ABC inference scheme, the inference accuracy of model parameters is higher for diverged sequences compared with closely related sequences (note that in these cases, the simulated true alignments were unaligned and realigned so that alignment errors are accounted for in this comparison). Thus, the benefit of additional information from the availability of more indel events in diverged sequences outweighs the possible harmful effect of decreased alignment reliability.

The RIM model established in this study is more elaborate than our previous model (SIM) that assumed equal attributes of insertions and deletions (Levy Karin et al. 2017). It was previously shown for both prokaryotes and eukaryotes that there is a deletion bias on sites which are assumed to evolve under neutral selection (Petrov et al. 1996; Ophir and Graur 1997; Mira et al. 2001; Zhang and Gerstein 2003; Van Passel et al. 2007; Kuo and Ochman 2009). Here we show, for a large variety of phylogenetic clades, that there is a deletion bias also for protein-coding sequences, which generally evolve under strong purifying selection, and that this bias is mainly due to high deletion rates, rather than due to longer deletion events. Of note, in our models, the estimated deletion rates are normalized by the substitution rate. Often, when comparing two organisms, one is inferred to have both higher deletion to substitution rate and higher substitution rate. Together, these two factors result in markedly different deletion dynamics, which may have impact on genome sizes (Petrov et al. 2000).

The indel models developed here have several limitations and there is still much room for more realistic modeling extensions. First, our results suggest that we are able to accurately infer indel model parameters for simulated data. However, empirical data are generated by processes that are likely more complicated than those assumed in the proposed models. How these model over-simplifications affect inference accuracy needs to be further studied. For example, both SIM and RIM assume that the indel parameters are uniform across the entire phylogeny. When highly diverged empirical data sets are analyzed, this assumption could be violated (indel heterotachy), and in this case, inferred model parameters may be a weighted average of few distinct indel dynamics. Similarly, errors in the topology or branch lengths of the assumed phylogenetic tree may bias the resulting inference. In addition, the inference scheme applied here assumes that the same indel dynamics equally characterize all positions within the input sequences. However, it was shown that the composition of amino acids in and around indels is significantly different from their composition across the entire sequence length, with enrichment of amino acids ADQEGPS and depletion of FMILYVWC (Chang and Benner 2004). Other studies also demonstrated that indel rates depend on the amino acid context (De La Chaux et al. 2007; Messer and Arndt 2007; Tanay and Siggia 2008; Kvikstad et al. 2009; Kvikstad and Duret 2014). Ideally, empirical context-dependent indel models should be developed and tested. In these models, the rate of insertions and the rate of deletions should each depend on the amino acid composition surrounding the indel site. Such models are expected to have a large number of free parameters, and thus resemble empirical amino acid replacement matrices such as JTT (Jones et al. 1992), WAG (Whelan and Goldman 2001), and LG (Le and Gascuel 2008). Accurate inference of the model parameters would require simultaneous analysis of a large amount of data, for example, the entire set of mammalian MSAs. High-quality genomic data are increasingly available and provide fertile ground for the development of such models. Of note, the ABC inference scheme described above relies on efficient simulators. To accelerate parameter inference, we implemented a simulator that generates indel events only and does not include substitution events. The above context-dependent models will necessitate simulating indel and substitution events simultaneously.

Another direction for future advance is to develop indel models that account for structural features of protein-coding genes. It is expected that different structural attributes do not share the same indel dynamics. For example, it was recently shown that for enhanced green fluorescent protein (eGFP), the packing density of a residue, as measured by the weighted contact number (Lin et al. 2008), considerably affects the probability that a single-residue deletion disrupts the protein's function (Jackson et al. 2017). Relative surface accessibility and secondary structure attributes were also found to affect this probability. Future indel models can explicitly account for such factors and should prove particularly useful, providing that the secondary or tertiary structures of a protein are available or can be accurately predicted.

Most commonly used alignment algorithms maximize a specific score and do not explicitly assume a stochastic Markov process. Recently, advances have been made in the development of statistical alignment methods, allowing simultaneous model parameter inference and alignment (Suchard and Redelings 2006; Nov**á**k et al. 2008; Bradley et al. 2009; Nute et al. 2019). Mikl**ó**s et al. (2004) and Levy Karin et al. (2019) have developed the long-indel model, in which both indels and substitutions evolve along a phylogenetic tree assuming a joint continuous-time Markov process. The rate of indels in this model depends on the indel length. Such a model was shown to better fit empirical data compared with previous models such as TKF91 (Thorne et al. 1991). However, it requires extensive computational time and it is currently limited to pairwise sequences. This model also assumes that deletion and insertion events have the same dynamics. The results of this study corroborate previous studies showing that for a large number of empirical data sets, insertion and deletion events are characterized by different evolutionary dynamics. Such considerations should be included in future statistical alignment methodologies.

An additional potential application of our methodology would be to characterize how indel dynamics vary among different proteins and protein domains, for example, it was previously suggested that ancient protein domains (Wolf et al. 2007) and highly conserved proteins (Ajawatanawong and Baldauf 2013) have a bias toward insertions and that essential proteins in bacteria and yeast experience more indel events than nonessential proteins (Chan et al. 2007). Finally, our methodology can be applied to quantify and test hypotheses regarding variation in indel dynamics in various genomics contexts, for example, in protein coding regions, long noncoding RNAs, introns, promotors, enhancers, near the telomeres, regions with high or low recombination rates, etc., and should provide statistically sound means to compare indel dynamics among genes and genomes across the tree of life.

## Materials and Methods

### Source Code and Implementation Details

The implementation of the algorithm presented here is called SpartaABC. It is implemented in C++ and Python. The source code is freely available at https://github.com/gilloe/SpartaABC (last accessed September 9, 2021). The scikit-learn Python package was used for machine learning. The SIM and RIM models, including the model selection schemes were added to the SpartaABC webserver: https://spartaabc.tau.ac.il/ (last accessed September 9, 2021; Ashkenazy et al. 2017). A Docker that enables installing and running a standalone version of SpartaABC can be downloaded from the webserver.

### Simulations

To characterize the performance of SpartaABC, we simulated sequences based on phylogenetic trees derived from empirical data sets. Specifically, we relied on 13 EggNOG (Huerta-Cepas et al. 2019) empirical data sets (supplementary table S2, Supplementary Material online), which were selected as they vary with respect to the tree topology, the number of sequences, the alignment lengths, and their level of divergence. Based on the phylogenetic tree of each of these 13 empirical data sets, we simulated 200 MSAs, to a total of 2,600 simulated data sets. Each of these 2,600 alignments was simulated with a different set of model parameters, sampled from the prior of RIM. Of note, the prior of all model parameters is identical between data sets except for the *R_L*, which depends on the length of the empirical alignment length (see New Approaches section). SpartaABC was then used to infer parameters for each of the 2,600 simulated data sets, and the Pearson correlation between the parameters used to simulate the data and those inferred using SpartaABC were reported. Figure 1 shows the results for all 200 simulated data sets derived from the empirical data set ENOG504B73R, whereas results for all simulations derived from the 13 empirical data sets are shown in supplementary table S2, Supplementary Material online.

Additional simulations were conducted for assessing the model-selection accuracy. We repeated the same simulations described above, however, this time, half of the simulations were generated under SIM and half under RIM. This resulted in a total of 1,300 SIM and 1,300 RIM data sets. The model-selection procedure described in the New Approaches section was used to determine which model best fits each of these 2,600 simulated data sets. Table 3 shows the results for all 200 simulated data sets derived from the empirical data set ENOG504B73R, whereas results regarding model selection for all simulations derived from the 13 empirical data sets are shown in supplementary table S3, Supplementary Material online.

### Analysis of Empirical Data Sets

The data used for the feature importance analyses are based on 13 EggNOG (Huerta-Cepas et al. 2019) data sets (supplementary fig. S3, Supplementary Material online). The data used to generate table 3 and figure 2 are based on the tree and MSA of EggNOG entry ENOG504B73R. The biological data sets, that is, the empirical phylogenetic trees and MSAs, were also downloaded from EggNOG and YIDB (Lopez and S**é**raphin 2000). Due to computational limitations, and in order that each taxonomic group will contain similar number of data sets, inclusion criteria were applied. Specifically, for the EggNOG data sets, we determined a minimal MSA length and a minimal number of species for each taxonomic group (supplementary table S4, Supplementary Material online). For YIDB data sets, we filtered MSAs with less than four species. In addition, when analyzing the empirical EggNOG and YIDB data sets, we filtered data sets in which the total branch lengths of the tree was smaller than 1.0, to avoid cases in which there are not enough indel events which are required for reliable estimation of model parameters. Such filtering was not performed in the simulations study.

## Supplementary Material

Supplementary data are available at Molecular Biology and Evolution online.

## Supplementary Material

msab266_Supplementary_Data

## References

[msab266-B1] Abadi S , Azouri D, Pupko T, Mayrose I. 2019. Model selection may not be a mandatory step for phylogeny reconstruction. Nat Commun. 10:934. 30804347 10.1038/s41467-019-08822-w PMC6389923

[msab266-B2] Ajawatanawong P , Baldauf SL. 2013. Evolution of protein indels in plants, animals and fungi. BMC Evol Biol. 13:140. 23826714 10.1186/1471-2148-13-140 PMC3706215

[msab266-B3] Anzai T , Shiina T, Kimura N, Yanagiya K, Kohara S, Shigenari A, Yamagata T, Kulski JK, Naruse TK, Fujimori Y, et al 2003. Comparative sequencing of human and chimpanzee MHC class I regions unveils insertions/deletions as the major path to genomic divergence. Proc Natl Acad Sci U S A. 100(13):7708–7713. 12799463 10.1073/pnas.1230533100 PMC164652

[msab266-B4] Ashkenazy H , Levy Karin E, Mertens Z, Cartwright RA, Pupko T. 2017. SpartaABC: a web server to simulate sequences with indel parameters inferred using an approximate Bayesian computation algorithm. Nucleic Acids Res. 45(W1):W453–W457. 28460062 10.1093/nar/gkx322 PMC5570005

[msab266-B5] Ashkenazy H , Penn O, Doron-Faigenboim A, Cohen O, Cannarozzi G, Zomer O, Pupko T. 2012. FastML: a web server for probabilistic reconstruction of ancestral sequences. Nucleic Acids Res. 40(Web Server issue):W580–W584. 22661579 10.1093/nar/gks498 PMC3394241

[msab266-B6] Beaumont MA , Zhang W, Balding DJ. 2002. Approximate Bayesian computation in population genetics. Genetics 162(4):2025–2035. 12524368 10.1093/genetics/162.4.2025 PMC1462356

[msab266-B7] Benner SA , Cohen MA, Gonnet GH. 1993. Empirical and structural models for insertions and deletions in the divergent evolution of proteins. J Mol Biol. 229(4):1065–1082. 8445636 10.1006/jmbi.1993.1105

[msab266-B8] Bradley RK , Roberts A, Smoot M, Juvekar S, Do J, Dewey C, Holmes I, Pachter L. 2009. Fast statistical alignment. PLoS Comput Biol. 5(5):e1000392. 19478997 10.1371/journal.pcbi.1000392 PMC2684580

[msab266-B9] Britten RJ. 2002. Divergence between samples of chimpanzee and human DNA sequences is 5%, counting indels. Proc Natl Acad Sci U S A. 99(21):13633–13635. 12368483 10.1073/pnas.172510699 PMC129726

[msab266-B10] Britten RJ , Rowen L, Williams J, Cameron RA. 2003. Majority of divergence between closely related DNA samples is due to indels. Proc Natl Acad Sci U S A. 100(8):4661–4665. 12672966 10.1073/pnas.0330964100 PMC153612

[msab266-B11] Cartwright RA. 2005. DNA assembly with gaps (Dawg): simulating sequence evolution. Bioinformatics. 21(Suppl 3):iii31–iii38. 16306390 10.1093/bioinformatics/bti1200

[msab266-B12] Cartwright RA. 2009. Problems and solutions for estimating indel rates and length distributions. Mol Biol Evol. 26(2):473–480. 19042944 10.1093/molbev/msn275 PMC2734402

[msab266-B13] Chan SK , Hsing M, Hormozdiari F, Cherkasov A. 2007. Relationship between insertion/deletion (indel) frequency of proteins and essentiality. BMC Bioinformatics. 8(1):227. 17598914 10.1186/1471-2105-8-227 PMC1925122

[msab266-B14] Chang MSS , Benner SA. 2004. Empirical analysis of protein insertions and deletions determining parameters for the correct placement of gaps in protein sequence alignments. J Mol Biol. 341(2):617–631. 15276848 10.1016/j.jmb.2004.05.045

[msab266-B15] Cliften P , Sudarsanam P, Desikan A, Fulton L, Fulton B, Majors J, Waterston R, Cohen BA, Johnston M. 2003. Finding functional features in Saccharomyces genomes by phylogenetic footprinting. Science 301(5629):71–76. 12775844 10.1126/science.1084337

[msab266-B16] De Jong WW , Rydén L. 1981. Causes of more frequent deletions than insertions in mutations and protein evolution. Nature 290(5802):157–159. 7207597 10.1038/290157a0

[msab266-B17] De La Chaux N , Messer PW, Arndt PF. 2007. DNA indels in coding regions reveal selective constraints on protein evolution in the human lineage. BMC Evol Biol. 7:191. 17935613 10.1186/1471-2148-7-191 PMC2151769

[msab266-B18] Fan Y , Wang W, Ma G, Liang L, Shi Q, Tao S. 2007. Patterns of insertion and deletion in mammalian genomes. Curr Genomics. 8(6):370–378. 19412437 10.2174/138920207783406479 PMC2671719

[msab266-B19] Fitch WM. 1973. Aspects of molecular evolution. Annu Rev Genet. 7:343–380. 4593308 10.1146/annurev.ge.07.120173.002015

[msab266-B20] Fletcher W , Yang Z. 2009. INDELible: a flexible simulator of biological sequence evolution. Mol Biol Evol. 26(8):1879–1888. 19423664 10.1093/molbev/msp098 PMC2712615

[msab266-B21] Gillespie DT. 1977. Exact stochastic simulation of coupled chemical reactions. J Phys Chem. 81(25):2340–2361.

[msab266-B22] Golenberg EM , Clegg MT, Durbin ML, Doebley J, Ma DP. 1993. Evolution of a noncoding region of the chloroplast genome. Mol Phylogenet Evol. 2(1):52–64. 8081547 10.1006/mpev.1993.1006

[msab266-B23] Graur D , Shuali Y, Li WH. 1989. Deletions in processed pseudogenes accumulate faster in rodents than in humans. J Mol Evol. 28(4):279–285. 2499684 10.1007/BF02103423

[msab266-B24] Gu X , Li WH. 1995. The size distribution of insertions and deletions in human and rodent pseudogenes suggests the logarithmic gap penalty for sequence alignment. J Mol Evol. 40(4):464–473. 7769622 10.1007/BF00164032

[msab266-B25] Huerta-Cepas J , Szklarczyk D, Heller D, Hernández-Plaza A, Forslund SK, Cook H, Mende DR, Letunic I, Rattei T, Jensen LJ, et al 2019. EggNOG 5.0: a hierarchical, functionally and phylogenetically annotated orthology resource based on 5090 organisms and 2502 viruses. Nucleic Acids Res. 47(D1):D309–D314. 30418610 10.1093/nar/gky1085 PMC6324079

[msab266-B26] Jackson EL , Spielman SJ, Wilke CO. 2017. Computational prediction of the tolerance to amino-acid deletion in green-fluorescent protein. PLoS One. 12(4):e0164905. 28369116 10.1371/journal.pone.0164905 PMC5378326

[msab266-B27] Jones DT , Taylor WR, Thornton JM. 1992. The rapid generation of mutation data matrices from protein sequences. Comput Appl Biosci. 8(3):275–282. 1633570 10.1093/bioinformatics/8.3.275

[msab266-B30] Katoh K , Standley DM. 2013. MAFFT multiple sequence alignment software version 7: improvements in performance and usability. Mol Biol Evol. 30(4):772–780. 23329690 10.1093/molbev/mst010 PMC3603318

[msab266-B31] Kozlov AM , Darriba D, Flouri T, Morel B, Stamatakis A. 2019. RAxML-NG: a fast, scalable and user-friendly tool for maximum likelihood phylogenetic inference. Bioinformatics 35(21):4453–4455. 31070718 10.1093/bioinformatics/btz305 PMC6821337

[msab266-B32] Kuhlwilm M , Han S, Sousa VC, Excoffier L, Marques-Bonet T. 2019. Ancient admixture from an extinct ape lineage into bonobos. Nat Ecol Evol. 3(6):957–965. 31036897 10.1038/s41559-019-0881-7

[msab266-B33] Kuo C-H , Ochman H. 2009. Deletional bias across the three domains of life. Genome Biol Evol. 1:145–152. 20333185 10.1093/gbe/evp016 PMC2817411

[msab266-B34] Kvikstad EM , Chiaromonte F, Makova KD. 2009. Ride the wavelet: a multiscale analysis of genomic contexts flanking small insertions and deletions. Genome Res. 19(7):1153–1164. 19502380 10.1101/gr.088922.108 PMC2704434

[msab266-B35] Kvikstad EM , Duret L. 2014. Strong heterogeneity in mutation rate causes misleading hallmarks of natural selection on indel mutations in the human genome. Mol Biol Evol. 31(1):23–36. 24113537 10.1093/molbev/mst185 PMC3879449

[msab266-B28] Levy Karin E , Rabin A, Ashkenazy H, Shkedy D, Avram O, Cartwright RA, Pupko T. 2015. Inferring indel parameters using a simulation-based approach. Genome Biol Evol. 7(12):3226–3238. 26537226 10.1093/gbe/evv212 PMC4700945

[msab266-B29] Levy Karin E , Shkedy D, Ashkenazy H, Cartwright RA, Pupko T. 2017. Inferring rates and length-distributions of indels using approximate Bayesian computation. Genome Biol Evol. 9(5):1280–1294. 28453624 10.1093/gbe/evx084 PMC5438127

[msab266-B36] Le SQ , Gascuel O. 2008. An improved general amino acid replacement matrix. Mol Biol Evol. 25(7):1307–1320. 18367465 10.1093/molbev/msn067

[msab266-B37] Levy Karin E , Ashkenazy H, Hein J, Pupko T. 2019. A simulation-based approach to statistical alignment. Syst Biol. 68(2):252–266. 30239957 10.1093/sysbio/syy059

[msab266-B38] Lin CP , Huang SW, Lai YL, Yen SC, Shih CH, Lu CH, Huang CC, Hwang JK. 2008. Deriving protein dynamical properties from weighted protein contact number. Proteins 72(3):929–935. 18300253 10.1002/prot.21983

[msab266-B39] Liu H , Zhang J. 2019. Yeast spontaneous mutation rate and spectrum vary with environment. Curr Biol. 29(10):1584–1591. 31056389 10.1016/j.cub.2019.03.054 PMC6529271

[msab266-B40] Lopez PJ , Séraphin B. 2000. YIDB: the Yeast Intron DataBase. Nucleic Acids Res. 28(1):85–86. 10592188 10.1093/nar/28.1.85 PMC102386

[msab266-B41] Lunter G. 2007. Probabilistic whole-genome alignments reveal high indel rates in the human and mouse genomes. Bioinformatics 23(13):i289–i296. 17646308 10.1093/bioinformatics/btm185

[msab266-B42] Messer PW , Arndt PF. 2007. The majority of recent short DNA insertions in the human genome are tandem duplications. Mol Biol Evol. 24(5):1190–1197. 17322553 10.1093/molbev/msm035

[msab266-B43] Miklós I , Lunter GA, Holmes I. 2004. A “long indel” model for evolutionary sequence alignment. Mol Biol Evol. 21(3):529–540. 14694074 10.1093/molbev/msh043

[msab266-B44] Mira A , Ochman H, Moran NA. 2001. Deletional bias and the evolution of bacterial genomes. Trends Genet. 17(10):589–596. 11585665 10.1016/s0168-9525(01)02447-7

[msab266-B45] Novák Á , Miklós I, Lyngsø R, Hein J. 2008. StatAlign: an extendable software package for joint Bayesian estimation of alignments and evolutionary trees. Bioinformatics 24(20):2403–2404. 18753153 10.1093/bioinformatics/btn457

[msab266-B46] Nute M , Saleh E, Warnow T. 2019. Evaluating statistical multiple sequence alignment in comparison to other alignment methods on protein data sets. Syst Biol. 68(3):396–411. 30329135 10.1093/sysbio/syy068 PMC6472439

[msab266-B47] Ogata H , Fujibuchi W, Kanehisa M. 1996. The size differences among mammalian introns are due to the accumulation of small deletions. FEBS Lett. 390(1):99–103. 8706839 10.1016/0014-5793(96)00636-9

[msab266-B48] Ophir R , Graur D. 1997. Patterns and rates of indel evolution in processed pseudogenes from humans and murids. Gene 205 (1–2):191–202. 9461394 10.1016/s0378-1119(97)00398-3

[msab266-B49] Pascarella S , Argos P. 1992. Analysis of insertions/deletions in protein structures. J Mol Biol. 224(2):461–471. 1560462 10.1016/0022-2836(92)91008-d

[msab266-B50] Petrov DA , Lozovskaya ER, Hartl DL. 1996. High intrinsic rate of DNA loss in Drosophila. Nature 384(6607):346–349. 8934517 10.1038/384346a0

[msab266-B51] Petrov DA , Sangster TA, Johnston JS, Hartl DL, Shaw KL. 2000. Evidence for DNA loss as a determinant of genome size. Science 287(5455):1060–1062. 10669421 10.1126/science.287.5455.1060

[msab266-B52] Pritchard JK , Seielstad MT, Perez-Lezaun A, Feldman MW. 1999. Population growth of human Y chromosomes: a study of y chromosome microsatellites. Mol Biol Evol. 16(12):1791–1798. 10605120 10.1093/oxfordjournals.molbev.a026091

[msab266-B53] Przeworski M. 2003. Estimating the time since the fixation of a beneficial allele. Genetics 164(4):1667–1676. 12930770 10.1093/genetics/164.4.1667 PMC1462667

[msab266-B54] Qian B , Goldstein RA. 2001. Distribution of indel lengths. Proteins Struct Proteins. 45(1):102–104. 10.1002/prot.1129 11536366

[msab266-B55] Robert CP , Cornuet J-M, Marin J-M, Pillai NS. 2011. Lack of confidence in approximate Bayesian computation model choice. Proc Natl Acad Sci U S A. 108(37):15112–15117. 21876135 10.1073/pnas.1102900108 PMC3174657

[msab266-B56] Saitou N , Ueda S. 1994. Evolutionary rates of insertion and deletion in noncoding nucleotide sequences of primates. Mol Biol Evol. 11(3):504–512. 8015443 10.1093/oxfordjournals.molbev.a040130

[msab266-B57] Shoemaker JS , Fitch WM. 1989. Evidence from nuclear sequences that invariable sites should be considered when sequence divergence is calculated. Mol Biol Evol. 6(3):270–289. 2622335 10.1093/oxfordjournals.molbev.a040550

[msab266-B58] Sisson SA. 2018. High-Dimensional ABC. In: Sisson SA, Fan Y, Beaumont MA, editors. Handbook of approximate Bayesian computation. Boston (MA): CRC Press. p. 213, 215.

[msab266-B59] Suchard MA , Redelings BD. 2006. BAli-Phy: simultaneous Bayesian inference of alignment and phylogeny. Bioinformatics 22(16):2047–2048. 16679334 10.1093/bioinformatics/btl175

[msab266-B60] Tallmon DA , Koyuk A, Luikart G, Beaumont MA. 2008. COMPUTER PROGRAMS: onesamp: a program to estimate effective population size using approximate Bayesian computation. Mol Ecol Resour. 8(2):299–301. 21585773 10.1111/j.1471-8286.2007.01997.x

[msab266-B61] Tanay A , Siggia ED. 2008. Sequence context affects the rate of short insertions and deletions in flies and primates. Genome Biol. 9(2):R37. 18291026 10.1186/gb-2008-9-2-r37 PMC2374710

[msab266-B62] Tavaré S. 1986. Some probabilistic and statistical problems in the analysis of DNA sequences. Am. Math. Soc. Lect. Math. Life Sci. 17:57–86.

[msab266-B63] Tavaré S , Balding DJ, Griffiths RC, Donnelly P. 1997. Inferring coalescence times from DNA sequence data. Genetics 145(2):505–518. 9071603 10.1093/genetics/145.2.505 PMC1207814

[msab266-B64] Thorne JL , Kishino H, Felsenstein J. 1991. An evolutionary model for maximum likelihood alignment of DNA sequences. J Mol Evol. 33(2):114–124. 1920447 10.1007/BF02193625

[msab266-B65] Tibshirani R. 1996. Regression shrinkage and selection via the lasso. J. R. Stat. Soc. Ser. B. 58(1):267–288.

[msab266-B66] Van Passel MWJ , Smillie CS, Ochman H. 2007. Gene decay in archaea. Archaea 2(2):137–143. 17350934 10.1155/2007/165723 PMC2686384

[msab266-B67] Vialle RA , Tamuri AU, Goldman N, Thorne J. 2018. Alignment modulates ancestral sequence reconstruction accuracy. Mol Biol Evol. 35(7):1783–1797. 29618097 10.1093/molbev/msy055 PMC5995191

[msab266-B68] Wetterbom A , Sevov M, Cavelier L, Bergström TF. 2006. Comparative genomic analysis of human and chimpanzee indicates a key role for indels in primate evolution. J Mol Evol. 63(5):682–690. 17075697 10.1007/s00239-006-0045-7

[msab266-B69] Whelan S , Goldman N. 2001. A general empirical model of protein evolution derived from multiple protein families using a maximum-likelihood approach. Mol Biol Evol. 18(5):691–699. 11319253 10.1093/oxfordjournals.molbev.a003851

[msab266-B70] Wolf Y , Madej T, Babenko V, Shoemaker B, Panchenko AR. 2007. Long-term trends in evolution of indels in protein sequences. BMC Evol Biol. 7:19. 17298668 10.1186/1471-2148-7-19 PMC1805498

[msab266-B71] Yang Z. 1994. Maximum likelihood phylogenetic estimation from DNA sequences with variable rates over sites: approximate methods. J Mol Evol. 39(3):306–314. 7932792 10.1007/BF00160154

[msab266-B72] Zhang Z , Gerstein M. 2003. Patterns of nucleotide substitution, insertion and deletion in the human genome inferred from pseudogenes. Nucleic Acids Res. 31(18):5338–5348. 12954770 10.1093/nar/gkg745 PMC203328

