# Supplementary material for: A Probabilistic Model for Indel Evolution: Differentiating Insertions from Deletions
Source: Mol Biol Evol. 2021 Sep 1;38(12):5769–81. doi: 10.1093/molbev/msab266 (PMC8662616; doi:10.1093/molbev/msab266)
Supplement: msab266_Supplementary_Data [file msab266_supplementary_data.pdf]

A probabilistic model for indel evolution: differentiating insertions from deletions

Supplementary Information

Gil Loewenthal[1], Dana Rapoport[1], Oren Avram[1], Asher Moshe[1], Elya Wygoda[1], Alon Itzkovitch[1], Omer Israeli[1], Dana Azouri[1,2], Reed A. Cartwright[3,4], Itay Mayrose[2], and Tal Pupko[1,†]

[1] The Shmunis School of Biomedicine and Cancer Research, George S. Wise Faculty of Life Sciences, Tel Aviv University, Tel Aviv 69978, Israel.

[2] School of Plant Sciences and Food Security, George S. Wise Faculty of Life Sciences, Tel Aviv University, Tel Aviv 69978, Israel.

[3] The Biodesign Institute, Arizona State University, Tempe, Arizona, USA.

[4] School of Life Sciences, Arizona State University, Tempe, Arizona, USA.

† To whom correspondence should be addressed:

Tal Pupko, Tel: +972 3 640 7693; Fax: +972 3 642 2046; E-mail: talp@tauex.tau.ac.il

Supplementary Tables

Table S1. **Computation of summary statistics and their corrections using machine-learning regression.** The summary statistics are derived from the alignment generated in the example provided in Figure S1. The value of the summary statistics from the true MSA (third column) are distorted after the alignment is un-aligned and re-aligned using MAFFT (fourth column). A regression model is used to correct for this bias (fifth column).

#	Summary statistic	From true MSA	From MAFFT MSA	After a regression-based correction
1	Total number of gap blocks in the alignment	16	11	12.1
2	Total number of unique gap blocks in the alignment	7	6	5.5
3	Average gap block length	3.3	4.1	5.4
4	Average unique gap block length	2.9	3.5	5.4
5	Number of gap blocks of length one	5	3	3.7
6	Number of gap blocks of length two	6	3	3.4
7	Number of gap blocks of length three	2	2	1.4
8	Number of gap blocks of length four or more	3	3	3.5

9	Alignment length	73	71	72.3
10	Minimum length of sequence in the alignment	55	55	56.7
11	Maximum length of sequence in the alignment	65	65	66.7
12	Number of MSA columns with zero gaps	53	52	54.3
13	Number of MSA columns with one gap	2	6	3.6
14	Number of MSA columns with two gaps	3	0	2.1
15	Number of MSA columns with n-1 gaps	15	13	12.2
16	Number of gaps of length one that appear only in one sequence	2	0	0.7
17	Number of gaps of length one that are shared between exactly two sequences	0	0	0
18	Number of gaps of length one that are shared between exactly n-1 sequences	1	1	1.1

19	Number of gaps of length two that appear only in one sequence	0	1	0.2
20	Number of gaps of length two that are shared between exactly two sequences	0	1	0.1
21	Number of gaps of length two that are shared between exactly n-1 sequences	2	0	1.0
22	Number of gaps of length three that appear only in one sequence	0	2	0.5
23	Number of gaps of length three that are shared between exactly two sequences	1	0	0.5
24	Number of gaps of length three that are shared between exactly n-1 sequences	0	0	0.0
25	Number of gaps of length at least four that appear only in one sequence	0	0	0.3
26	Number of gaps of length at least four that	0	0	0.4

	are shared between exactly two sequences			
27	Number of gaps of length at least four that are shared between exactly n-1 sequences	1	1	0.8

Table S2. Performance of SpartaABC in simulations based on 13 EggNOG v5.0 benchmark datasets (http://eggnog5.embl.de/#/app/home). Each benchmark dataset dictates the phylogenetic tree (and thus also the number of sequences) and the distribution of root length used for simulations (which depends on the alignment length). Repeated simulations along the phylogenetic tree of each dataset, allowed us to compare true and inferred parameters and compute the correlation between the two sets. The slope was derived from linear regression, setting the intercept to zero.

Group	EggNOG accession (id)	Sum of branch lengths [aa replacements per site]	MSA average length [aa]	Number of sequences	Parameter	R^2	Slope
Bacillus	ENOG50 1ZD87	5.50	491	10	RL	0.95	0.93
					R_I	0.86	0.84
					R_D	0.82	0.84
					A_I	0.60	0.65
					A_D	0.70	0.67
Brassicales	ENOG50 3HQ0R	2.39	1,691	18	RL	0.97	0.96
					R_I	0.80	0.72
					R_D	0.74	0.71
					A_I	0.65	0.63
					A_D	0.52	0.48
Chlorophyta	ENOG50 34J2H	0.97	794	10	RL	0.98	0.93
					R_I	0.78	0.70
					R_D	0.67	0.64
					A_I	0.41	0.34
					A_D	0.33	0.28
Ciliophora	ENOG50 3ZBCQ	4.07	705	8	RL	0.97	0.95
					R_I	0.84	0.81
					R_D	0.83	0.83
					A_I	0.64	0.65
					A_D	0.52	0.54
						0.96	0.93

Drosophilidae	ENOG50 45P83	11.21	1,020	24	RL		
					R_I	0.84	0.77
					R_D	0.91	0.90
					A_I	0.81	0.78
					A_D	0.64	0.54
Escherichia	ENOG50 3XQHM	0.25	332	6	RL	0.94	0.81
					R_I	0.35	0.34
					R_D	0.35	0.31
					A_I	0.14	0.12
					A_D	0.08	0.08
P. aeruginosa	ENOG50 1YEJ1	8.10	1,250	14	RL	0.14	0.14
					R_I	0.84	0.81
					R_D	0.82	0.80
					A_I	0.50	0.52
					A_D	0.54	0.55
Primates	ENOG50 4M5J3	0.06	422	13	RL	0.98	0.91
					R_I	0.28	0.29
					R_D	0.25	0.27
					A_I	0.04	0.07
					A_D	0.05	0.06
Rhabditida	ENOG50 40WJ9	2.82	936	11	RL	0.96	0.92
					R_I	0.89	0.84
					R_D	0.86	0.83
					A_I	0.64	0.56
					A_D	0.59	0.52
Rhizobiaceae	ENOG50 4B73R	30.15	817	129	RL	0.93	0.87
					R_I	0.87	0.82
					R_D	0.92	0.90
					A_I	0.81	0.78
					A_D	0.71	0.65
Rodentia	ENOG50 4Q6VV	0.24	2,341	16	RL	0.97	0.92
					R_I	0.56	0.50

					R_D	0.56	0.48
					A_I	0.29	0.25
					A_D	0.34	0.22
Saccharomycetaceae	ENOG50 3RZ5D	23.00	869	20	RL	0.78	0.80
					R_I	0.76	0.75
					R_D	0.78	0.81
					A_I	0.69	0.75
					A_D	0.50	0.51
Tenericutes	ENOG50 3WTGV	9.73	821	10	RL	0.30	0.35
					R_I	0.71	0.76
					R_D	0.72	0.76
					A_I	0.39	0.50
					A_D	0.41	0.54

Table S3. Accuracy of the model selection classification. The accuracy (number of cases for which the model selection classifier correctly identified the generative model) analysis is based on 2,600 simulated datasets, derived from 13 empirical datasets (100 SIM and 100 RIM simulated datasets based on each empirical dataset).

Group	EggNOG accession (id)	SIM Accuracy (%)	RIM Accuracy (%)
Bacillus	ENOG501ZD87	86	64
Brassicales	ENOG503HQ0R	67	64
Chlorophyta	ENOG5034J2H	88	60
Ciliophora	ENOG503ZBCQ	85	61
Drosophilidae	ENOG5045P83	96	66
Escherichia	ENOG503XQHM	33	74
P. aeruginosa	ENOG501YEJ1	79	76
Primates	ENOG504M5J3	54	63
Rhabditida	ENOG5040WJ9	81	62
Rhizobiaceae	ENOG504B73R	98	77
Rodentia	ENOG504Q6VV	59	68
Saccharomycetaceae	ENOG503RZ5D	81	78
Tenericutes	ENOG503WTGV	81	68

Table S4. **Average MSA length, number of sequences and total branch length for each empirical taxonomic group.** In parenthesis, inclusion thresholds per each group. For example, only alignment longer than 800 amino acids, and trees with at least 8 sequences, and total branch lengths larger than 1.0 amino-acid replacements per site were considered for *Bacillus*.

Group	MSA length	Number of sequences	Total branch lengths measured in amino-acid replacements per site
Bacillus	1234.4 (800)	36.4 (8)	19.0 (1)
Escherichia	661.7 (300)	10.5 (5)	2.7 (1)
P. aeruginosa	1031.9 (700)	10.2 (4)	3.8 (1)
Rhizobiaceae	1092.0 (800)	38.6 (8)	10.6 (1)
Staphylococcaceae	781.3 (500)	23.3 (5)	7.0 (1)
Tenericutes	1205.0 (700)	22.7 (4)	14.8 (1)
Vibrionales	1016.1 (700)	28.4 (7)	10.4 (1)
Brassicales	1990.7 (1500)	21.0 (7)	3.8 (1)
Chlorophyta	1807.2 (1000)	11.8 (10)	8.3 (1)
Ciliophora	1780.3 (800)	12.8 (8)	9.0 (1)
Drosophilidae	2306.2 (1500)	11.3 (10)	3.1 (1)
Primates	2395.4 (1500)	16.2 (10)	1.9 (1)
Rhabditida	1732.1 (1000)	13.6 (10)	6.4 (1)
Rodentia	1586.2 (1000)	16.5 (15)	2.6 (1)
Saccharomycetaceae	1450.2 (1000)	16.8 (15)	16.2 (1)

Supplementary Figures

Inputs:

(1) Tree

(Species1:0.4,(Species2:0.15,(Species3:0.15, Species4:0.2):0.25):0.45);

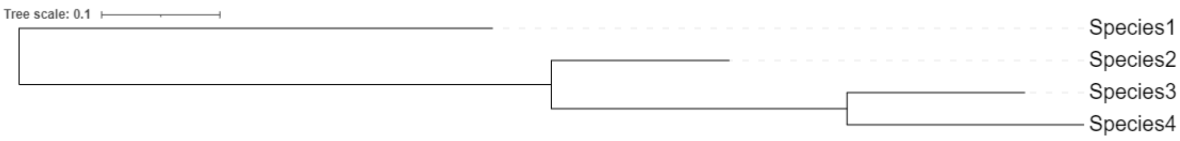

(2) Simulations Parameters

Parameter name	Value
RL	54
R_I	0.024
R_D	0.003
A_I	1.40
A_D	1.45

(b) Simulation of indels along the tree

Simulated MSA

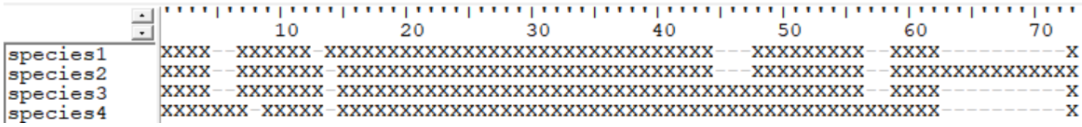

(c) Simulating substitutions, needed for computing distortions introduced by alignment methods.

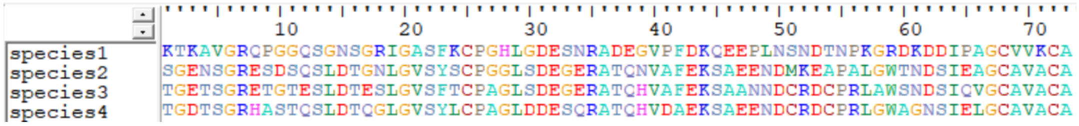

(d) Superimposing indels and substitutions.

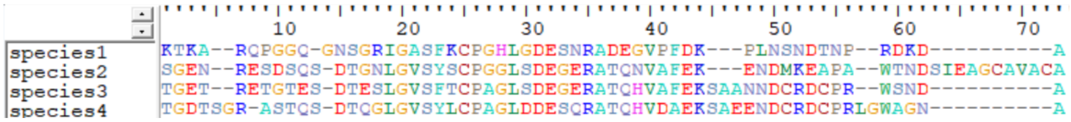

(e) The alignment is unaligned and then re-aligned with MAFFT

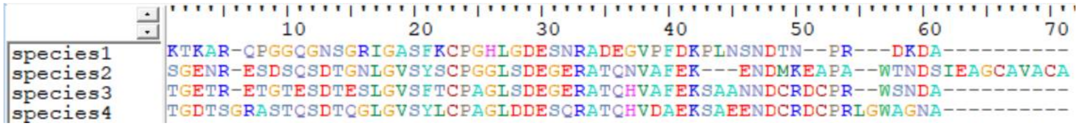

Figure S1. Example of a simulation. (a) To generate a benchmark dataset, a phylogenetic tree with branch length is needed, as well as a set of true model parameters; (b) We implemented our own simulator that efficiently simulates alignments based on the Gillispie algorithm, using the input model parameters and a phylogenetic tree. Our simulator does not generate substitutions, substantially accelerating its speed. Shown is the output of a simulation; (c) For learning how MAFFT distorts summary statistics, we need to generate 200 MSAs with substitutions. This is done by first generating an INDELIBLE alignment without gaps in which the alignment length is the same as the alignment generated by our simulator; (d) The two alignments are superimposed to generate an alignment that includes both indels and substitutions; (e) The alignment is un-aligned and re-aligned using MAFFT, thus generating a benchmark alignment that better reflects empirical alignments. The phylogenetic tree and MSAs are displayed using itol (Letunic and Bork 2019) and BioEdit (Hall 1999), respectively.

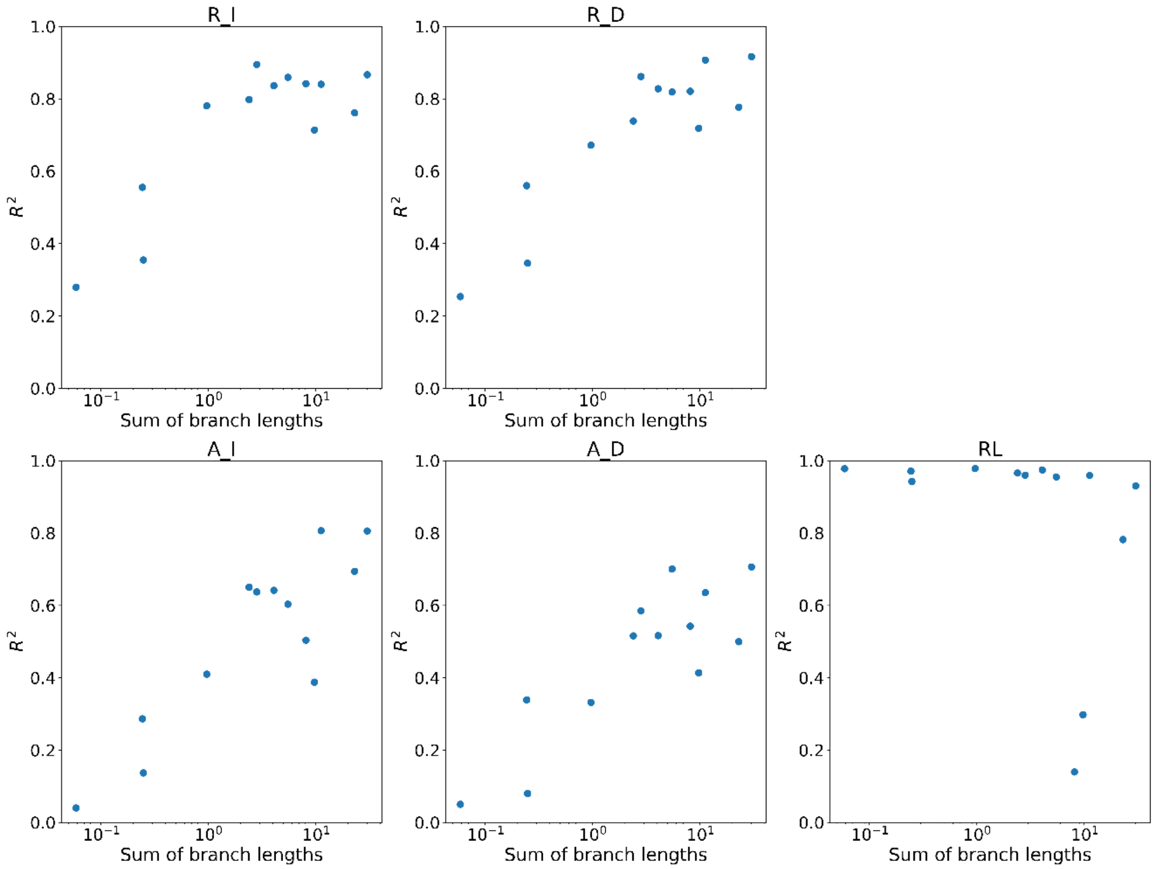

Figure S2. Scatter plots of the R^2 of the simulation set (from table S2) versus the sum of branch lengths for the inferred parameters (R_I, R_D, A_I, A_D, and RL).

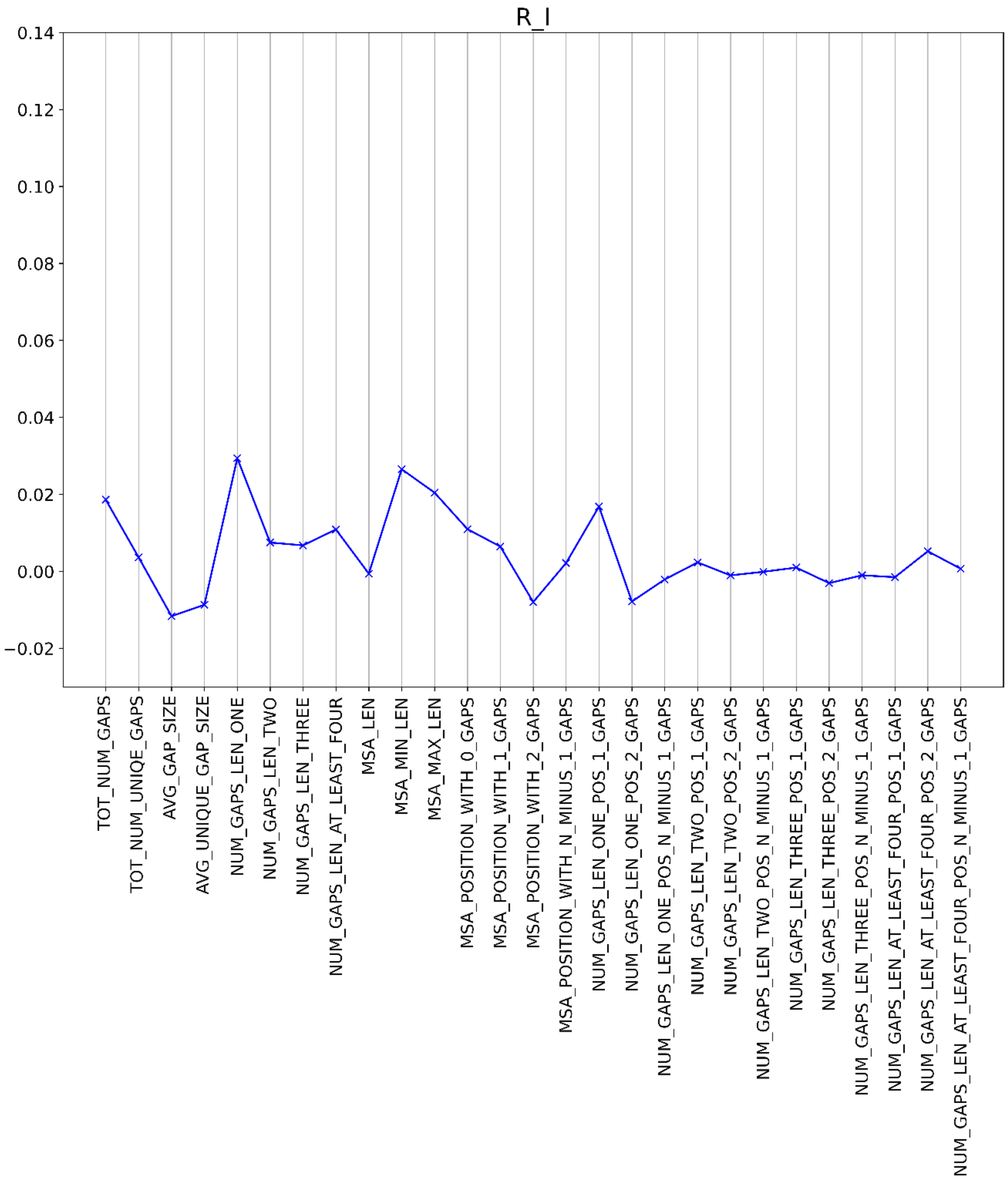
R_I
0.14
0.12
0.10
0.08
0.06
0.04
0.02
0.00
−0.02
TOT_NUM_GAPS
TOT_NUM_UNIQE_GAPS
AVG_GAP_SIZE
AVG_UNIQUE_GAP_SIZE
NUM_GAPS_LEN_ONE
NUM_GAPS_LEN_TWO
NUM_GAPS_LEN_THREE
NUM_GAPS_LEN_AT_LEAST_FOUR
MSA_LEN
MSA_MIN_LEN
MSA_MAX_LEN
MSA_POSITION_WITH_0_GAPS
MSA_POSITION_WITH_1_GAPS
MSA_POSITION_WITH_2_GAPS
MSA_POSITION_WITH_N_MINUS_1_GAPS
NUM_GAPS_LEN_ONE_POS_1_GAPS
NUM_GAPS_LEN_ONE_POS_2_GAPS
NUM_GAPS_LEN_ONE_POS_N_MINUS_1_GAPS
NUM_GAPS_LEN_TWO_POS_1_GAPS
NUM_GAPS_LEN_TWO_POS_2_GAPS
NUM_GAPS_LEN_TWO_POS_N_MINUS_1_GAPS
NUM_GAPS_LEN_THREE_POS_1_GAPS
NUM_GAPS_LEN_THREE_POS_2_GAPS
NUM_GAPS_LEN_THREE_POS_N_MINUS_1_GAPS
NUM_GAPS_LEN_AT_LEAST_FOUR_POS_1_GAPS
NUM_GAPS_LEN_AT_LEAST_FOUR_POS_2_GAPS
NUM_GAPS_LEN_AT_LEAST_FOUR_POS_N_MINUS_1_GAPS

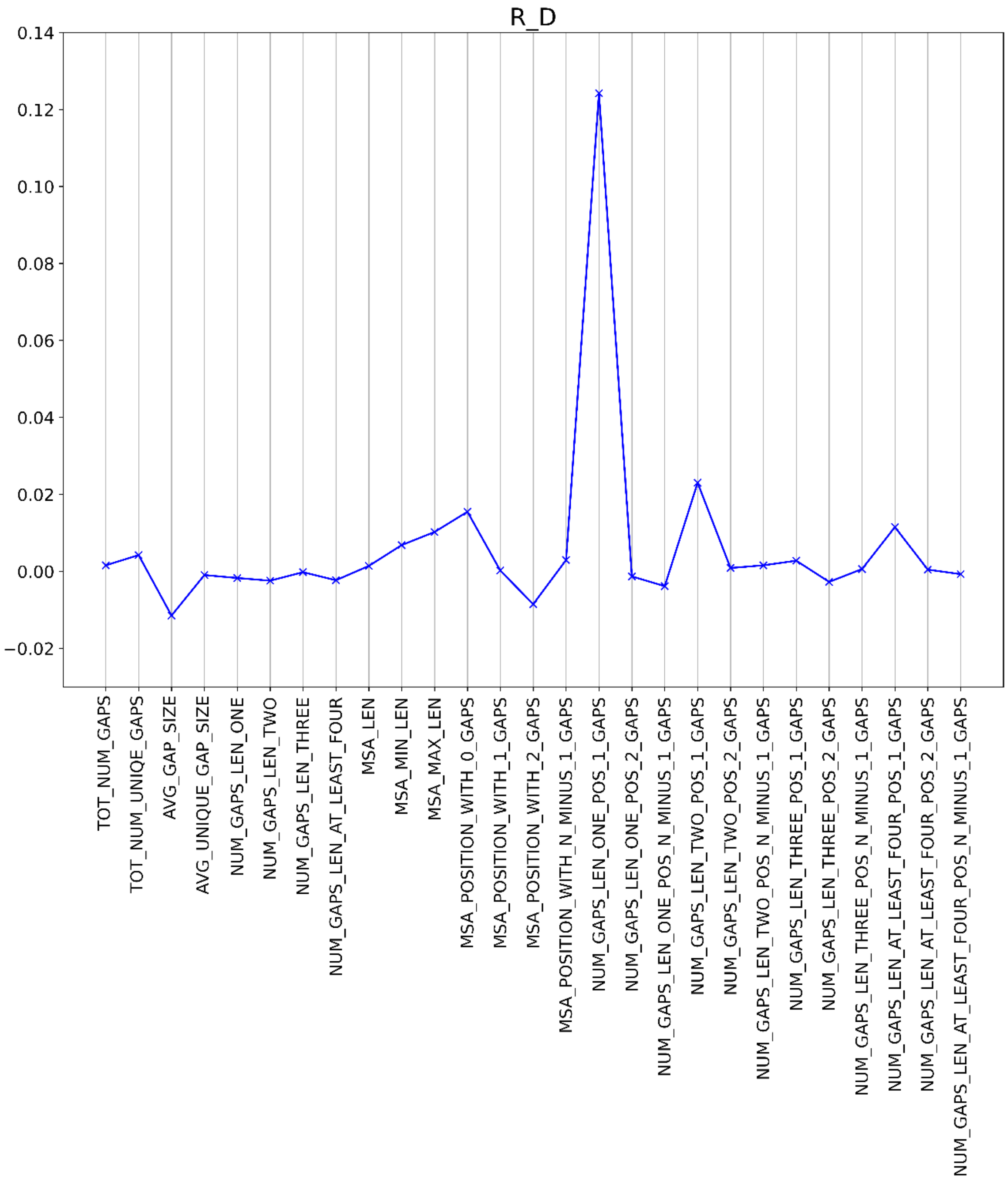

R_D
0.14
0.12
0.10
0.08
0.06
0.04
0.02
0.00
−0.02
TOT_NUM_GAPS
TOT_NUM_UNIQE_GAPS
AVG_GAP_SIZE
AVG_UNIQUE_GAP_SIZE
NUM_GAPS_LEN_ONE
NUM_GAPS_LEN_TWO
NUM_GAPS_LEN_THREE
NUM_GAPS_LEN_AT_LEAST_FOUR
MSA_LEN
MSA_MIN_LEN
MSA_MAX_LEN
MSA_POSITION_WITH_0_GAPS
MSA_POSITION_WITH_1_GAPS
MSA_POSITION_WITH_2_GAPS
MSA_POSITION_WITH_N_MINUS_1_GAPS
NUM_GAPS_LEN_ONE_POS_1_GAPS
NUM_GAPS_LEN_ONE_POS_2_GAPS
NUM_GAPS_LEN_ONE_POS_N_MINUS_1_GAPS
NUM_GAPS_LEN_TWO_POS_1_GAPS
NUM_GAPS_LEN_TWO_POS_2_GAPS
NUM_GAPS_LEN_TWO_POS_N_MINUS_1_GAPS
NUM_GAPS_LEN_THREE_POS_1_GAPS
NUM_GAPS_LEN_THREE_POS_2_GAPS
NUM_GAPS_LEN_THREE_POS_N_MINUS_1_GAPS
NUM_GAPS_LEN_AT_LEAST_FOUR_POS_1_GAPS
NUM_GAPS_LEN_AT_LEAST_FOUR_POS_2_GAPS
NUM_GAPS_LEN_AT_LEAST_FOUR_POS_N_MINUS_1_GAPS

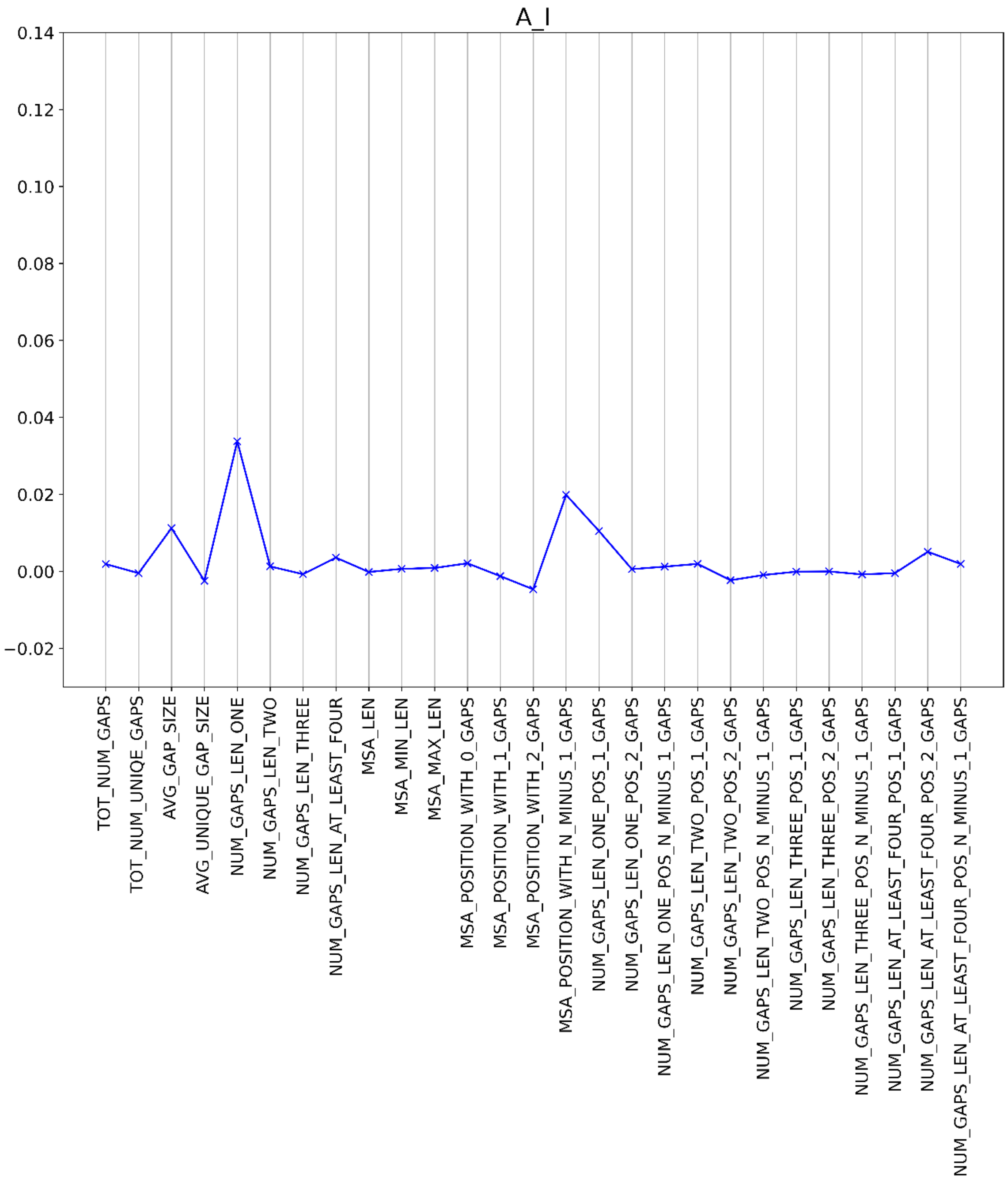
A_I
0.14
0.12
0.10
0.08
0.06
0.04
0.02
0.00
−0.02
TOT_NUM_GAPS
TOT_NUM_UNIQE_GAPS
AVG_GAP_SIZE
AVG_UNIQUE_GAP_SIZE
NUM_GAPS_LEN_ONE
NUM_GAPS_LEN_TWO
NUM_GAPS_LEN_THREE
NUM_GAPS_LEN_AT_LEAST_FOUR
MSA_LEN
MSA_MIN_LEN
MSA_MAX_LEN
MSA_POSITION_WITH_0_GAPS
MSA_POSITION_WITH_1_GAPS
MSA_POSITION_WITH_2_GAPS
MSA_POSITION_WITH_N_MINUS_1_GAPS
NUM_GAPS_LEN_ONE_POS_1_GAPS
NUM_GAPS_LEN_ONE_POS_2_GAPS
NUM_GAPS_LEN_ONE_POS_N_MINUS_1_GAPS
NUM_GAPS_LEN_TWO_POS_1_GAPS
NUM_GAPS_LEN_TWO_POS_2_GAPS
NUM_GAPS_LEN_TWO_POS_N_MINUS_1_GAPS
NUM_GAPS_LEN_THREE_POS_1_GAPS
NUM_GAPS_LEN_THREE_POS_2_GAPS
NUM_GAPS_LEN_THREE_POS_N_MINUS_1_GAPS
NUM_GAPS_LEN_AT_LEAST_FOUR_POS_1_GAPS
NUM_GAPS_LEN_AT_LEAST_FOUR_POS_2_GAPS
NUM_GAPS_LEN_AT_LEAST_FOUR_POS_N_MINUS_1_GAPS

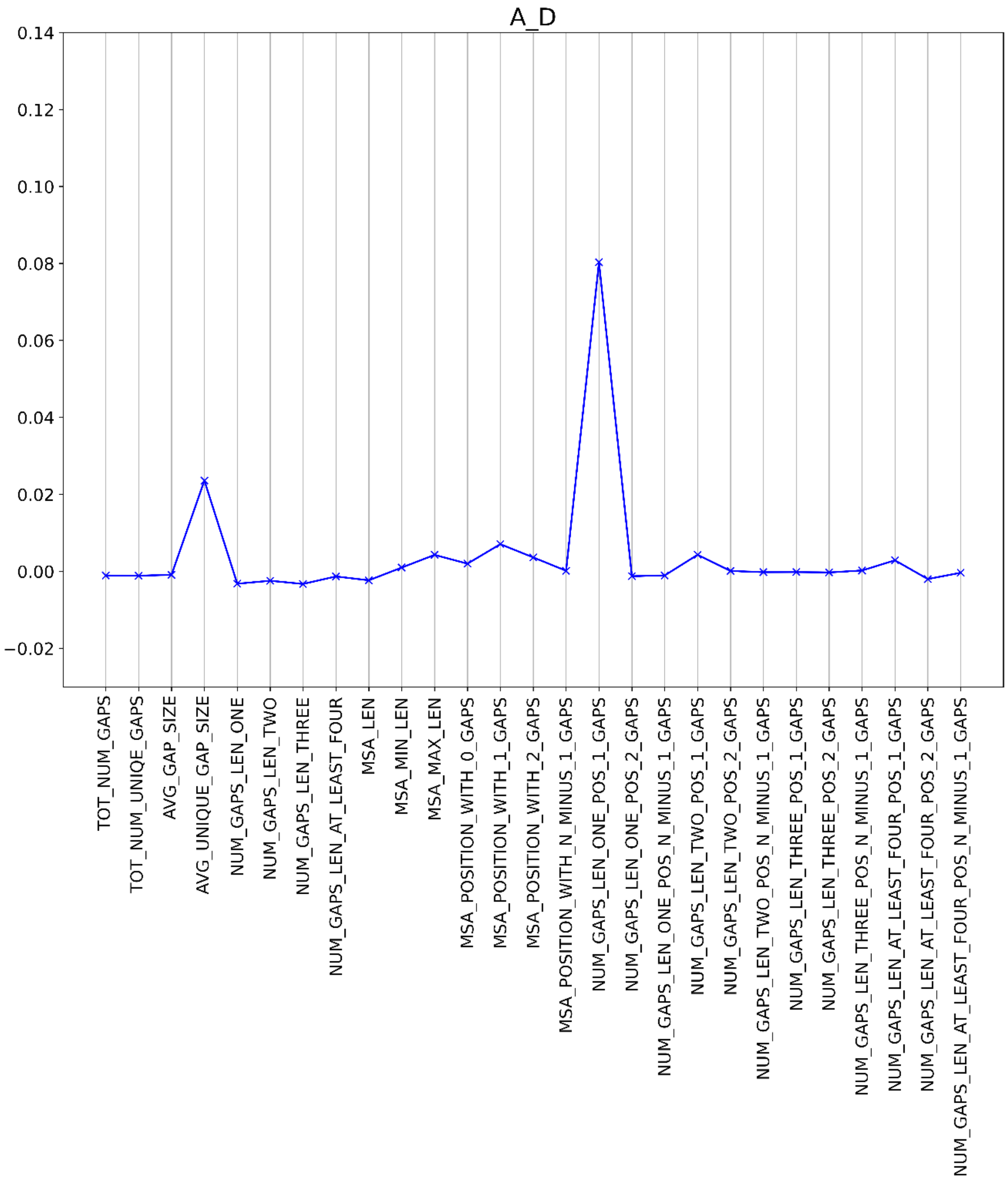

A_D
0.14
0.12
0.10
0.08
0.06
0.04
0.02
0.00
−0.02
TOT_NUM_GAPS
TOT_NUM_UNIQE_GAPS
AVG_GAP_SIZE
AVG_UNIQUE_GAP_SIZE
NUM_GAPS_LEN_ONE
NUM_GAPS_LEN_TWO
NUM_GAPS_LEN_THREE
NUM_GAPS_LEN_AT_LEAST_FOUR
MSA_LEN
MSA_MIN_LEN
MSA_MAX_LEN
MSA_POSITION_WITH_0_GAPS
MSA_POSITION_WITH_1_GAPS
MSA_POSITION_WITH_2_GAPS
MSA_POSITION_WITH_N_MINUS_1_GAPS
NUM_GAPS_LEN_ONE_POS_1_GAPS
NUM_GAPS_LEN_ONE_POS_2_GAPS
NUM_GAPS_LEN_ONE_POS_N_MINUS_1_GAPS
NUM_GAPS_LEN_TWO_POS_1_GAPS
NUM_GAPS_LEN_TWO_POS_2_GAPS
NUM_GAPS_LEN_TWO_POS_N_MINUS_1_GAPS
NUM_GAPS_LEN_THREE_POS_1_GAPS
NUM_GAPS_LEN_THREE_POS_2_GAPS
NUM_GAPS_LEN_THREE_POS_N_MINUS_1_GAPS
NUM_GAPS_LEN_AT_LEAST_FOUR_POS_1_GAPS
NUM_GAPS_LEN_AT_LEAST_FOUR_POS_2_GAPS
NUM_GAPS_LEN_AT_LEAST_FOUR_POS_N_MINUS_1_GAPS

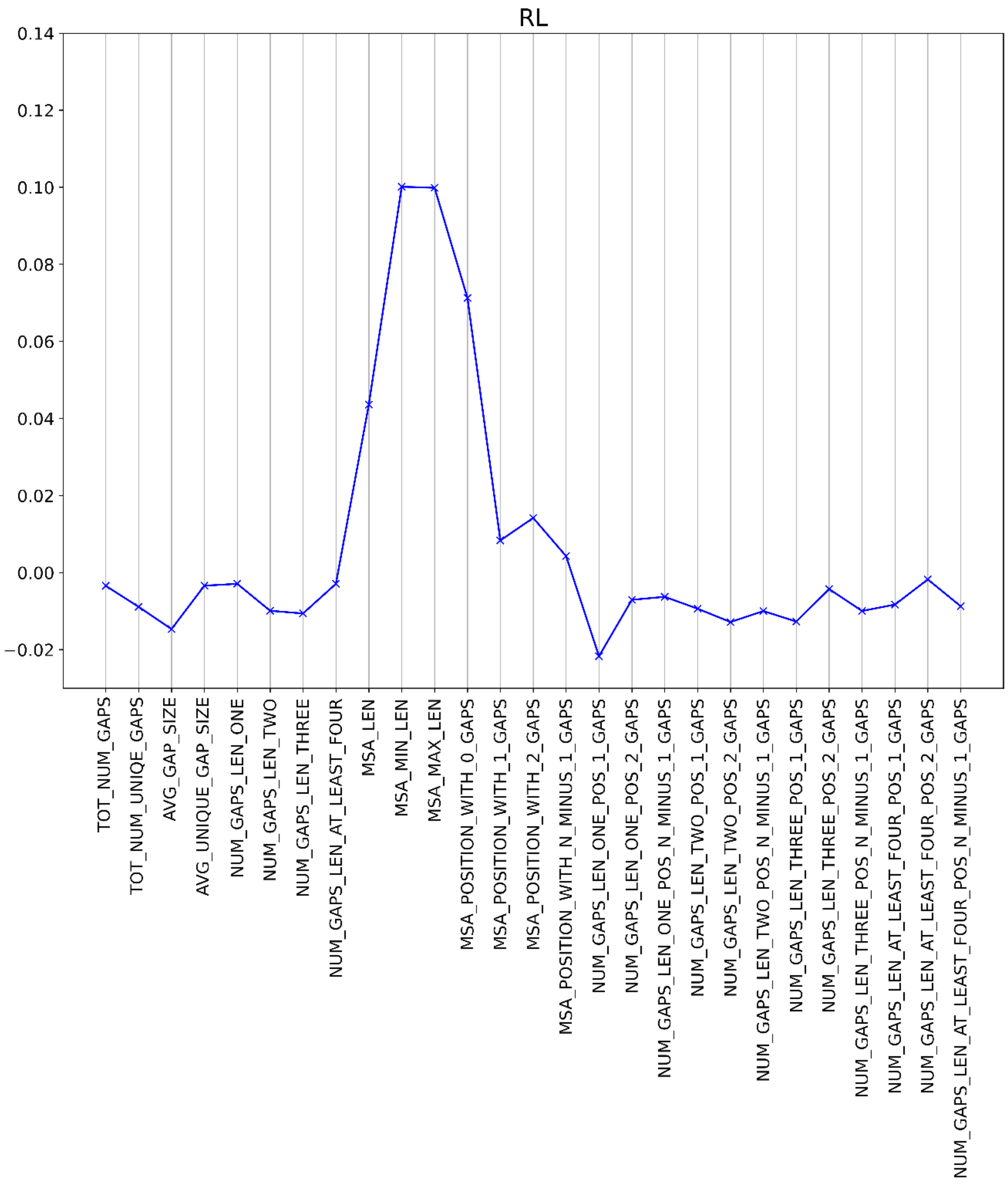

Figure S3. Feature importance. Each of the five panels corresponds to a different model parameter. Shown are the reduction of accuracy when excluding each summary statistic relative to using all the summary statistics. The results shown are an average of the datasets in table S2.

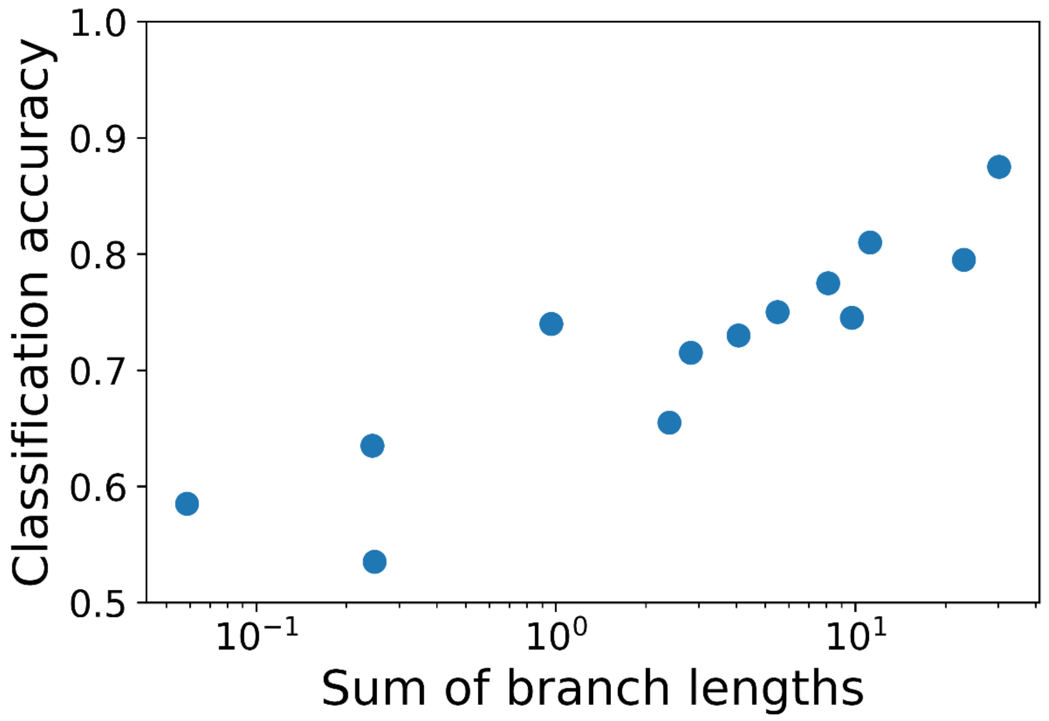

Figure S4. Classification accuracy as a function of the sum of branch lengths. Each dot represents classification accuracy over 200 simulations (100 SIM and 100 RIM). In each dot, the 200 simulations were generated along a phylogenetic tree of one of the 13 empirical datasets provided in table S3.

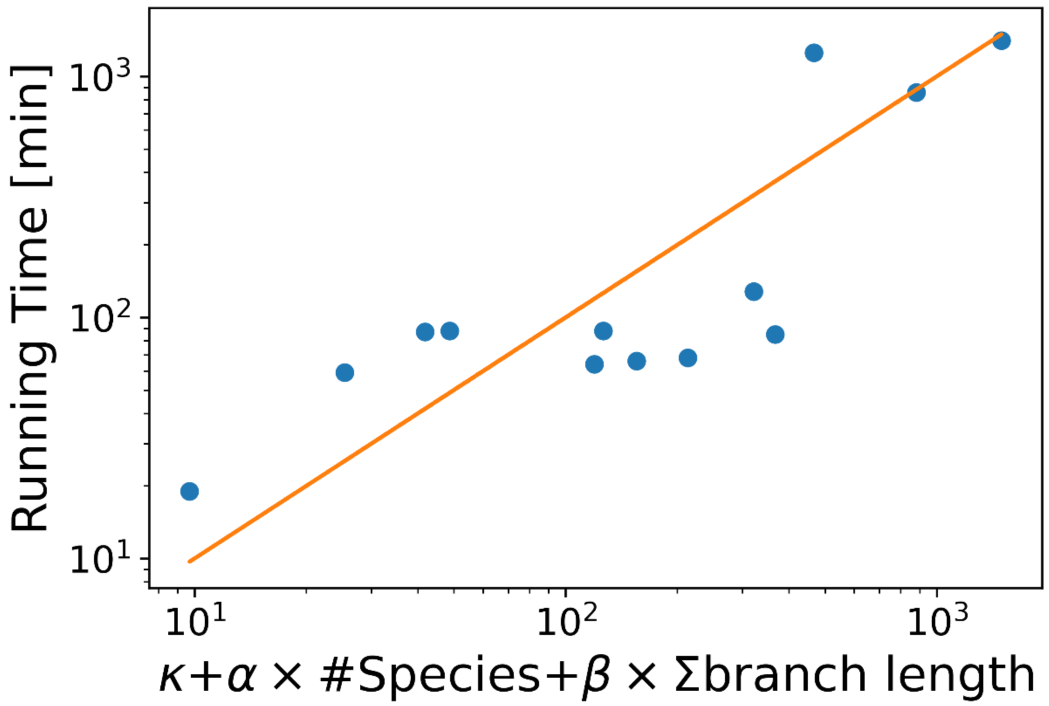

Figure S5. Running times are correlated to a linear combination of sum of branch lengths and number of species. $R^2 = 0.73$, P < 2e-4, $running\ time = 3.2 \times \#\ species + 36.4 \times sum\ of\ branch\ lengths - 18.7$. The sum of branch length and number of species for each accession number is given in Table S2. The data for this graph (EggNOG accession id, running times in minutes): (ENOG501ZD87,68), (ENOG503HQ0R,88), (ENOG5034J2H,88), (ENOG503ZBCQ,66), (ENOG5045P83,1254), (ENOG503XQHM,19), (ENOG501YEJ1,128), (ENOG504M5J3,59), (ENOG5040WJ9,64), (ENOG504B73R,1409), (ENOG504Q6VV,87), (ENOG503RZ5D,857), (ENOG503WTGV,85).

References

Hall, T.A. (1999). BIOEDIT: a user-friendly biological sequence alignment editor and analysis program for Windows 95/98/ NT. Nucleic Acids Symp. Ser. 41:95–98.

Letunic, I., and Bork, P. (2019). Interactive Tree of Life (iTOL) v4: Recent updates and new developments. Nucleic Acids Res. 47:W256–W259.
